# D3/Penta 21 clinical trial design: a randomised non-inferiority trial with nested drug licensing substudy to assess dolutegravir and lamivudine fixed dose formulations for the maintenance of virological suppression in children with HIV-1 infection, aged 2 to 15 years

**DOI:** 10.1016/j.cct.2024.107540

**Published:** 2024-04-16

**Authors:** Anna Turkova, Man K. Chan, Cissy Kityo, Adeodata R. Kekitiinwa, Philippa Musoke, Avy Violari, Ebrahim Variava, Moherndran Archary, Tim R. Cressey, Suwalai Chalermpantmetagul, Kanokkorn Sawasdichai, Pradthana Ounchanum, Suparat Kanjanavanit, Sakulrat Srirojana, Ussanee Srirompotong, Steven Welch, Alasdair Bamford, Cristina Epalza, Clàudia Fortuny, Angela Colbers, Eleni Nastouli, Simon Walker, Dan Carr, Magda Conway, Moira J Spyer, Nazia Parkar, Iona White, Alessandra Nardone, Margaret J Thomason, Rashida A Ferrand, Carlo Giaquinto, Deborah Ford, on behalf of the D3 trial team

**Affiliations:** 1 Medical Research Council Clinical Trials Unit at University College London, United Kingdom; 2 Joint Clinical Research Centre, Kampala, Uganda; 3 Baylor College of Medicine, Kampala, Uganda; 4 Makerere University–Johns Hopkins University Research Collaboration, Kampala, Uganda; 5 Perinatal HIV Research Unit, University of the Witwarsrand, Johannesburg, South Africa; 6 Department of Paediatrics and Children Health, King Edward VIII Hospital, Enhancing Care Foundation, University of KwaZulu-Natal, Durban, South Africa; 7 AMS-IRD PHPT Research Collaboration, Faculty of Associated Medical Sciences, Chiang Mai University, Chiang Mai, Thailand; 8 Prapokklao Hospital, Chantaburi, Thailand; 9 Chiangrai Prachanukroh Hospital, Chiang Rai, Thailand; 10 Nakornping Hospital, Chiang Mai, Thailand; 11 Kalasin Hospital, Kalasin, Thailand; 12 Khon Kaen Hospital, Khon Kaen, Thailand; 13 Heartlands Hospital, University Hospitals Birmingham NHS Foundation Trust, Birmingham, UK; 14 Great Ormond Street Hospital for Children NHS Foundation Trust, London, United Kingdom; 15 University College London Great Ormond Street Institute of Child Health, London, United Kingdom; 16 Instituto de Investigación Sanitaria Hospital 12 de Octubre (imas12), Madrid, Spain; 17 Infectious Diseases Department, Institut de Recerca Sant Joan de Déu, Sant Joan de Déu Children's Hospital, Barcelona, Spain; 18 Department of Surgery and Medico-Surgical Specialties, Faculty of Medicine and Health Sciences, Universitat de Barcelona, Barcelona, Spain; 19 Department of Pharmacy, Radboud Institute for Medical InnovationHealth Sciences, Radboud University Medical Center, Nijmegen, Netherlands; 20 University College London Hospitals NHS Trust, Advanced Pathogen Diagnostics Unit, London, United Kingdom; 21 Centre for Health Economics, University of York, Heslington, York, United Kingdom; 22 Department of Molecular and Clinical Pharmacology, University of Liverpool, United Kingdom; 23 Fondazione Penta ETS, Padova, Italy; 24 London School of Hygiene and Tropical Medicine, United Kingdom; 25 University of Padova, Department of Women and Child Health, Padova, Italy

**Keywords:** Randomized control trial, HIV, children, adolescents, dolutegravir/lamivudine, two-drug therapy

## Abstract

**Background:**

There is increasing interest in utilising two-drug regimens for HIV treatment with the goal of reducing toxicity and improve acceptability. The D3 trial evaluates the efficacy and safety of DTG/3TC in children and adolescents and includes a nested pharmacokinetics(PK) substudy for paediatric drug licensing.

**Methods:**

D3 is an ongoing open-label, phase III, 96-week non-inferiority randomised controlled trial(RCT) conducted in South Africa, Spain, Thailand, Uganda and the United Kingdom. D3 has enrolled 386 children aged 2-<15 years, virologically suppressed for ≥6 months, with no prior treatment failure. Participants were randomised 1:1 to receive DTG/3TC or DTG plus two nucleoside reverse transcriptase inhibitors(NRTIs), stratified by region, age (2-<6, 6-<12, 12-<15 years) and DTG use at enrolment (participants permitted to start DTG at enrolment). The primary outcome is confirmed HIV-1 RNA viral rebound ≥50 copies/mL by 96-weeks. The trial employs the Smooth Away From Expected(SAFE) non-inferiority frontier, which specifies the non-inferiority margin and significance level based on the observed event risk in the control arm. The nested PK substudy evaluates WHO weight-band-aligned dosing in the DTG/3TC arm.

**Discussion:**

D3 is the first comparative trial evaluating DTG/3TC in children and adolescents. Implications of integrating a PK substudy and supplying data for prompt regulatory submission, were carefully considered to ensure the integrity of the ongoing trial. The trial uses an innovative non-inferiority frontier for the primary analysis to allow for a lower-than-expected confirmed viral rebound risk in the control arm, while ensuring interpretability of results and maintaining the planned sample size in an already funded trial.

## Background

The landscape of HIV treatment is shifting. With improved survival and the need for lifelong antiretroviral therapy (ART), there is growing emphasis on optimizing treatment safety and quality of life while ensuring high effectiveness and affordability. This has led to the exploration of new approaches, such as two-drug regimens (2DR) instead of the standard three-drug regimens (3DR). Several adult trials have evaluated a switch to dolutegravir(DTG) plus lamivudine (3TC) 2DR (DTG/3TC) in adults who are virologically suppressed^1–15^ (Table 1), consistently showing non-inferiority of DTG/3TC compared to 3DRs in maintaining virological suppression. Reassuringly, efficacy of DTG/3TC has also been demonstrated in adults who are ART-naive,^16–24^ a population considered to be at a greater risk of treatment failure (Table 1). A network meta-analysis of 4 randomized controlled trials (RCTs) including >10,000 patients who were ART-naive demonstrated similar efficacy and safety of DTG/3TC compared with 3DRs over 48 weeks.^25^

M184V/I mutations markedly reduce HIV susceptibility to 3TC.^28^ A recent systematic review and meta-analysis examined the efficacy of DTG/3TC 2DR in virologically suppressed adults with historical or archived pre-switch M184V/I mutations in RCTs and real-world studies.^29^ Overall, virological failures in patients with pre-switch M184V/I were low, with 7/186 (4%) patients experiencing failure at 96 weeks in real-world studies and 0/34 (0%) in RCTs, respectively. No treatment-emergent resistance mutations were reported.

Two systematic reviews summarised real-world evidence for the DTG/3TC effectiveness in nearly 5000 adults. High rates of virological suppression (83% to 100%) were observed in both treatment-experienced and treatment-naïve groups.^30,31^ Treatment-emergent resistance was reported in one patient.^31^ Overall discontinuation rates due to adverse events were low (2 to 8%), with neuropsychiatric adverse events being the most common.^31^

There are limited data on DTG/3TC treatment in children and adolescents. There are concerns that adolescents have poorer adherence than adults,^32^ and 2DR treatment may perform worse in this population. DANCE is an ongoing single arm trial evaluating DTG/3TC in ART-naïve adolescents. At 96 weeks 22/25 (88%) were suppressed. No new safety concerns were observed compared to the established safety profile in adults.^26,27^ There are no comparative studies of DTG/3TC in children or adolescents. Current treatment guidelines, including guidelines issued by the World Health Organization, the United States Department of Health and Human Services and the European AIDS Clinical Society, do not yet recommend using DTG/3TC in children.

We describe here an ongoing RCT evaluating whether switching to DTG/3TC in children and adolescents can maintain similar virological suppression to standard 3DR ART while reducing the burden of lifelong ART and potential ART toxicities.

## Methods

### Objective/hypothesis

**Figure 1:**
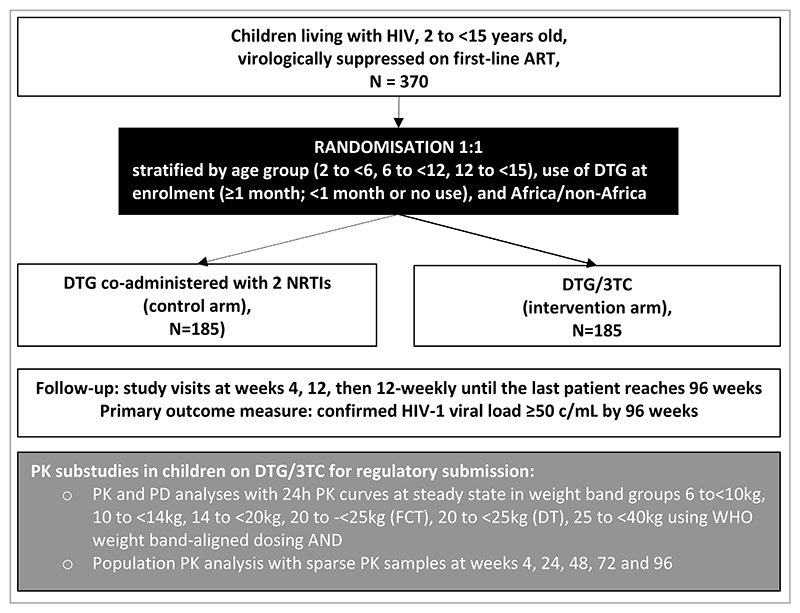
Trial Schema ART = antiretroviral therapy; DT= dispersible tablet; DTG/3TC = dolutegravir and lamivudine given as a fixed dose combination; FCT = film-coated tablet; NRTI = nucleoside reverse transcriptase inhibitors; PK = pharmacokinetic; PD = pharmacodynamic; WHO = World Health Organization

The D3 trial is comparing DTG/3TC, given as fixed dose combinations (FDCs), with a DTG plus 2 NRTIs in children and adolescents living with HIV who were virologically suppressed <50 copies/mL (c/mL) prior to switch (Figure 1). The primary hypothesis is that DTG/3TC will provide non-inferior virological suppression compared with DTG plus 2 NRTIs over 96 weeks.

### Study design, number of children, randomisation and follow-up

The D3 trial was initially designed to evaluate the efficacy and safety of DTG/3TC versus any standard-of-care 3DR. Between design and trial opening, ODYSSEY demonstrated that DTG provided superior efficacy and safety in children compared with other anchor drugs (mainly efavirenz and boosted protease inhibitors);^33^ consequently, children began to transition to DTG-based ART globally. The D3 control arm was therefore amended to DTG plus 2 NRTIs to ensure DTG/3TC would be compared to standard-of-care likely to remain relevant at the end of the trial.

The D3 trial is an ongoing, open-label, randomised non-inferiority trial, open in Spain, South Africa, Thailand, Uganda and the UK. Children aged 2 to <15 years were randomised 1:1 to DTG/3TC (intervention arm) or DTG plus 2 NRTIs (control arm). Children were permitted to start DTG at randomisation in either arm. Randomisation was stratified by age (2-<6; 6-<12; 12-<15), use of DTG at enrolment (≥1 month; <1 month or no use), and region (Africa; non-Africa). The computer-generated randomisation list was prepared using permuted blocks with variable size to reduce predictability of the next allocated treatment and incorporated securely into the trial database, concealed from local staff. Allocation for each patient was made automatically through the web-enabled database after eligibility has been confirmed by site staff.

A total of 386 children were enrolled over 16 months. A pharmacokinetic and safety substudy is nested in the intervention arm to provide timely data for licensing of DTG/3TC FDCs for children.

### Study population

The trial enrolled children, who were virologically suppressed for ≥6 months and had no history of treatment failure. Previous substitutions for toxicity, simplification, changes in guidelines or drug availability were allowed (Table 2).

### Treatment of patients

Children randomised to **the control arm** receive DTG plus 2 NRTIs, in line with WHO and European guidelines, recommending DTG as a preferred first-line anchor drug.^35,36^

Children randomised to **the DTG/3TC arm** receive novel paediatric dispersible tablets (DT) and adult film-coated tablet (FCT) of DTG/3TC dosed using WHO weight bands and provided by ViiV Healthcare (Table 3). DTG and 3TC doses for all weight bands, except 20-<25kg, were selected based on WHO recommendations for the individual components.^36^ Children 20-<25 kg either receive 6 DTs or one FCT, dependent on study site. The dose of 3TC at 300mg in the FCT is 1.3 times higher than the currently recommended dose for children 20-<25 kg; this dose is being investigated in the trial based on an acceptable safety profile of 3TC^37^ and potential patient preference for one FCT.

### Primary and secondary outcomes

The primary outcome is confirmed viral rebound (defined as the first of two consecutive HIV-1 RNA≥50 c/mL) by week 96. This outcome was chosen as an objective and clinically relevant measure of the loss of virological suppression. Secondary outcomes are listed in Table 4.

### Sample size

Non-inferiority of DTG/3TC will be assessed by the difference between the trial arms in the estimated proportion of participants with confirmed viral rebound by week 96.

When the D3 trial was designed, it was estimated that 370 participants (185 per arm) would provide 80% power to exclude a fixed non-inferiority margin of 10% for the difference in the the primary endpoint, assuming a 12% risk of confirmed viral rebound by 96 weeks in both arms, 10% loss to follow-up and a two-sided α of 0.05. The assumptions for the sample size calculation were made based on BREATHER results^38^ on efavirenz-based regimens in children and young people.

To ensure interpretability of results in the event of a lower-than-estimated risk of viral rebound in the control arm, we will implement the innovative Smooth Away From Expected (SAFE) frontier.^39^ This frontier defines the non-inferiority margin and significance level based on the observed confirmed viral rebound risk in the control arm, while preserving power and controlling for type 1 error (Appendix A, Figure S1).

Provided that the observed viral rebound risk in the control arm is ≥9%, a 95% two-sided confidence interval will be computed for the difference in viral rebound and a 10% non-inferiority margin will be used. If the observed viral rebound in the control arm is <9%, a 99% two-sided confidence interval will be computed; the non-inferiority margin will depend on the control event rate (Figure S1). If the upper bound of the respective confidence interval is no higher than the selected non-inferiority margin, then the null hypothesis will be rejected and DTG/3TC will be declared non-inferior to control.

### Recruitment and follow-up

D3 recruitment was conducted in outpatient clinics, where significant numbers of children living with HIV are treated. The trial aimed to enrol at least half of participants aged <12 years, to ensure enough young children received DTG/3TC FDC for safety evaluation. Due to rapid initial recruitment, primarily in the heavier weight bands, enrolment of children ≥25kg was capped in Africa and Thailand to ensure the PK substudy was able to fully recruit across all weight bands. Recruitment was not capped in Spain and the UK, due to the small numbers of potential participants in these countries.

Participants will be followed up until the last enrolled participant reaches 96 weeks; each participant will attend an end-of-trial visit at ≥96 weeks from enrolment and within ±6 weeks of the last participant reaching 96 weeks.

### Study procedures

All participants were seen at screening, enrolment, 4 and 12 weeks and will continue to be seen every 12 weeks until the end of follow-up (Appendix A, Table S1).

**HIV-1 viral load (VL)** was measured at the sites at the screening visit to confirm eligibility, and is measured in real time in follow-up at weeks 24, 48, 96 and 48-weekly thereafter (more frequently if recommended in local guidelines) (Appendix A, Table S1). Provision of more frequent than routinely done VL tests would likely alter patient management, meaning trial results would not apply to most of sub-Saharan Africa where routine monitoring is 6-12 monthly. Targeted real-time testing is also performed for suspected treatment failure.

Stored plasma samples are used for retrospective VL testing at all other timepoints. A combination of real-time and retrospective VL results are used to inform the trial endpoints and for review by the Data Monitoring Committee (DMC), which is meeting 6-monthly while the data are collected for the licensing submission, and then at least annually. The results on the stored samples are not returned to treating clinicians to inform clinical care.

The trial will utilise Next Generation Sequencing (NGS) for **HIV drug resistance testing**, using saved buffy coat samples at baseline and NGS on buffy coat samples together with traditional Sanger sequencing on plasma samples at the time of virological rebound (Appendix A, Table S1). The methodology developed in preparation for the D3 trial is expected to offer a sensitive tool for detecting mutations even at low level and an easy to implement assay in diverse settings.^40^

### Questionnaires

The trial utilises participant/carer questionnaires to evaluate participant adherence to treatment and acceptability of study medicines, mood and sleep, suicidal ideation and behaviour and health-related quality of life (Appendix A, Figures S2-S5). All questionnaires have been translated to local languages.

### Criteria for discontinuing or modifying allocated interventions

Participants on DTG/3TC who have two consecutive real-time VLs ≥50 c/mL should switch to a 3DR within 12 weeks of the second VL ≥50 c/mL. The choice of the 3DR is at the discretion of the site clinician and may depend on the results of resistance tests (if available) and the availability of age-appropriate antiretroviral formulations. The choice of the new ART regimen can be discussed with the trial physician. Participants in the control arm with 2 consecutive real time VLs ≥50 c/mL are managed as per national guidelines.

Protocol version 2.0 (used when the trial opened) required participants on DTG/3TC, who became pregnant, to change to DTG-based 3DR for the duration of pregnancy and breastfeeding. ViiV Healthcare’s approach to the management of pregnant participants has changed in line with the WHO’s call to action,^41^ and protocol version 3.0 (awaiting approvals) offers pregnant participants on DTG/3TC the option to either stay on DTG/3TC (with additional informed consent) or to change to a DTG-based 3DR. Additional real-time VL tests in the third trimester will be provided in both trial arms to maintain comparability of arms, and to ensure treatment enhancement can be implemented for any participant on DTG/3TC with viral rebound. Pregnant participants in the control arm continue their current 3DR.

For participants, requiring rifampicin treatment (e.g. for tuberculosis), weight-appropriate daily DTG doses are given twice-daily i.e. doubled (this applies to both arms; participants in the DTG/3TC arm can stay on DTG/3TC 2DR). Participants with newly diagnosed hepatitis B should be started on tenofovir-containing ART, if available; otherwise, they are managed on an individual basis. DTG is discontinued if participants develop liver injury (see liver stopping criteria and considerations for re-start in the Protocol, Section 5.8). If a patient experiences a moderate or high risk of suicidality during the trial, discontinuation of DTG is also considered.

In the control arm, ART changes may include a switch to DTG-based fixed dose ART combinations (FDC) or a change of NRTI backbone for treatment simplification. Other changes to ART are minimised unless unavoidable and in the best interests of the participant, e.g. due to use of concomitant medication or toxicity.

### Adverse event reporting and harms

Adverse events (AEs) of all severity grades are collected. AEs are graded using the Division of AIDS Table for Grading the Severity of Adult and Pediatric Adverse Events, corrected version 2.1 – July 2017.^42^ Neutrophil grading is based on NIH Paediatric toxicity tables^43^ and WHO 2010 recommendations for antiretroviral therapy in infants and children,^44^ recognising the lower normal levels in African populations. Suspected cases of drug-induced liver injury, possible suicidality-related adverse events, ABC hypersensitivity and pregnancies constitute notable events. Both serious adverse events (SAEs) and notable events are reported to the CTUs within expedited timelines. SAEs are reported to regulatory agencies as per national requirements.

### Strategies to improve adherence

Adherence is checked by pill count, short nurse-administered adherence questions and adherence questionnaires, completed by children (if they are deemed to be old enough) or their carers. All the trial sites provide adherence support as per standard of care.

### Data management

The trial database is programmed in OpenClinica and access is controlled. Worksheets and electronic case research forms (eCRFs) were created based on data collection requirements in the trial protocol. To protect participants’ confidentiality, participants were assigned a trial identification number and a random three-letter code. The trial database has programmed checks for eligibility, range checks for laboratory values and missing data checks. Additional consistency checks are performed by the trial statisticians. Adverse events (diagnoses) are coded using Medical Dictionary for Regulatory Activities.^45^

### Statistical analysis plan

For **the primary analysis**, the two treatment arms (DTG/3TC and control) will be compared in the intention-to-treat population, defined as all randomised participants excluding those with a major eligibility violation. To allow for censoring, the survival curve for each combination of strata and randomised arm will be calculated using a Cox model adjusting for stratification factors and randomised arm. The average cumulative failure function for each randomised arm will be estimated as a weighted average of the corresponding stratum-specific cumulative failure functions with weights equal to the prevalence of that stratum in the whole intention-to-treat population.^46^ The difference in the probability of virological rebound between the DTG/3TC and control arm will be estimated by the average difference between the cumulative failure functions at week 96. A 2-sided bias-corrected 95% or 99% confidence interval (selected as per Table 5) for the difference in the probability of virological rebound by week 96 (DTG/3TC – control) will be calculated with bootstrap standard errors. DTG/3TC will be considered non-inferior to DTG-based 3DR if the upper limit of the confidence interval of the difference DTG/3TC-control is less than the selected non-inferiority margin (Table 5). If non-inferiority is demonstrated, we will test for superiority of DTG/3TC versus DTG-based 3DR.

For analysis of **the primary endpoint and other virological outcomes**, except for the FDA snapshot analysis (Appendix A, Table S2), multiple imputation of missing HIV-1 RNA measurements at scheduled visits will be applied if either of the following criterium is met: 5% of all HIV-1 RNA measurements at scheduled visits are missing OR 10% of confirmatory HIV-1 RNA measurements are missing.

The cumulative probability of virological rebound by week 48 (**secondary outcome** measure) will be estimated similarly to the primary outcome. **A secondary analysis** will be done in the per protocol population, censoring follow-up after a participant makes a non-permitted treatment change to ART (including a change of ART component(s) for lack of efficacy, AE, pregnancy, protocol deviation or time off allocated regimen for ≥7 days for patient/carer decision) or stops treatment for >31 days. Other **secondary analyses** will evaluate treatment effects by the randomisation stratification factors. **Other secondary outcome measures** will be compared for superiority between the DTG/3TC and control arms using appropriate statistical methods in the intention-to-treat population (see Protocol, Section 9.6).

### Substudies

**An Intensive Pharmacokinetic (PK) and safety substudy** is nested in the trial to support regulatory approvals of DTG/3TC formulations for children. The substudy is conducted in the intervention arm only and aims to evaluate the pharmacokinetics, safety, tolerability and antiviral activity of DTG/3TC in virologically suppressed children aged ≥2 years and weighing <40kg using WHO weight band-aligned dosing. The substudy planned to recruit ≥8 children with evaluable PK curves per WHO weight-band and formulations (Table 3); and ≥14 children per age group 2 to <6 years and ≥6 years. Seven plasma samples were taken at least 7 days after starting DTG/3TC regimen (and ≥21 days after starting DTG) over 24 hours (t=0 (prior to observed dosing), 1, 2, 3, 4, 6 and 24h post-dosing). The substudy fully recruited in approximately 12 months.

Other trial substudies include Population Pharmacokinetics, Pharmacogenetics and Pharmacodynamics, Tuberculosis-Dolutegravir Pharmacokinetics, Once-weekly Isoniazid plus Rifapentine and Dolutegravir Pharmacokinetics, Virology and Health Economics (Appendix A).

### Trial oversight

The sponsor of the trial is Fondazione Penta ETS who has overall accountability. The sponsor has delegated set-up activities, management, monitoring, analysis and reporting of the trial to the MRC CTU at UCL. The Program for HIV Prevention and Treatment (PHPT) clinical trial unit in Thailand is responsible for trial implementation at clinical sites in the PHPT network in Thailand.

The Trial Steering Committee (TSC) provides overall supervision for the trial and consists of majority independent members. The Data Monitoring Committee (DMC) is formed of independent clinical trial experts who review interim analyses of accumulating data by trial arm. The DMC will advise the TSC if the trial should be stopped for safety reasons or if a definitive answer is reached earlier than the scheduled end of the trial. The Trial Management group (TMG) is a responsible for management of the trial. Members include the Chief Investigator, Sponsor Representatives Principal Investigators and other lead investigators at sites, substudy leads and members of MRC CTU at UCL and PHPT. At MCRC CTU at UCL a Trial Management Team (TMT) conducts the day-to-day running of the trial.

### Patient and public involvement

The trial team engages with existing patient liaison groups at sites, including Youth Trial Boards (YTBs) in South Africa, Uganda and the UK and local Community Advisory Boards. YTB model was developed in the ODYSSEY trial.^47^ YTBs consist of young individuals aged 14 to 19 years, who are supported by YTB coordinators. YTB members contribute to the design of patient information materials, participate in discussions about the trial and results interpretation and promote trial findings within their communities. Two representatives of the YTBs are included in the TSC, as non-voting independent members, to provide a voice and input during the trial.

### Current status of the trial

The first patient was enrolled in the trial in Thailand on 11 May 2022. Within a year, the intensive PK substudy had been fully recruited; and comprises 82 children who consented to PK, including 74 who completed PK visits successfully. In the main trial, 386 participants were enrolled in the sites in South Africa, Spain, Thailand, Uganda and UK by 28 Sep 2023.

### Considerations for provision of regulatory data for licensing during an ongoing trial

As described, the D3 trial is an academic-led trial with follow-up to 96 weeks after the last participant was recruited and a 96-week primary outcome; the trial is likely to report in early 2026. The PK and safety substudy includes 82 participants who consented to PK within the DTG/3TC arm (∼45% of DTG/3TC participants).

The regulatory submission which will be made by ViiV Healthcare will include follow-up to the last PK participant reaching 48 weeks. It will comprise all PK data, clinical and laboratory safety data, VL test results and participant acceptability data to end of substudy follow-up, and (to 48 weeks only) resistance results.

Data which will contribute to the submission, except VL and resistance data, are being provided 3-monthly to ViiV Healthcare. The DMC are meeting 6-monthly to coincide with alternate data transfers, such that they are in a position to respond, based on their review of comparative data across the whole trial, should ViiV Healthcare raise any safety concerns. Prior to the final data submission, substudy VL and resistance data will only be reviewed at ViiV Healthcare by statistics and programming teams, and virology experts who are not directly involved in the D3 trial. As per standard practice, these data are not being shared with the trial clinicians, to avoid conclusions being drawn about the efficacy of DTG/3TC based on limited early data. Data will be reviewed prior to submission by the ViiV Healthcare D3 team, but results will be kept confidential until the trial’s primary endpoint is reported.

## Discussion

D3 is the only paediatric randomised trial evaluating the 2DR combination of DTG and 3TC. Using a non-inferiority design, D3 aims to assess whether DTG/3TC can achieve comparable virological suppression to standard DTG-based 3DR in children and adolescents, while reducing potential toxicities and avoiding excess of treatment-emerging resistance.

**Children and adolescents living with HIV are a special population** who can benefit from 2DR HIV treatment. On average, they remain on ART for longer than adults, placing them at a higher risk of accumulating long-term toxicities and experiencing treatment fatigue. Additionally, children undergo rapid growth, pubertal and neurodevelopmental maturation, and therefore may benefit from reduced exposure to drugs in different ways to adults. On the other hand, adolescents may struggle more than adults with adherence to treatment, and on reduced therapy, they may be more susceptible to virological failure and emergence of drug resistance, which can compromise their future treatment options. The 96-week RCT data will provide the necessary evidence as to whether DTG/3TC can be used safely in virologically suppressed children, without increasing the risk of viral rebound and emerging resistance.

To ensure the **relevance of findings to diverse settings**, including sub-Saharan Africa where most of children with HIV live, and VL monitoring is less frequent than in other regions, the trial uses a pragmatic approach for real-time VL testing aligned with national guidelines. Additionally, retrospective VL testing of stored samples is performed to determine the trial's virological outcomes and for review by the DMC, who oversee participant safety. For confirmed viral rebound outcomes, we use the first HIV-1 RNA measure to minimise impact of different schedules for routine VL monitoring across sites. The trial also addresses the commonly cited reasons for not using DTG/3TC in low- or middle-income countries, including the need for co-treatment of TB, management of pregnancies, and newly diagnosed cases of hepatitis B. By incorporating strategies to manage these situations, the trial ensures that children and adolescents receive appropriate care when these events occur.

D3 is a non-inferiority trial which aims to demonstrate that the new treatment’s efficacy is not unacceptably lower than standard of care. The choice of the non-inferiority margin is critical to determine the acceptable loss of efficacy. If a fixed margin is used, its appropriateness is likely to depend on the control event risk being close to expected; where this is not the case, power and interpretability of results can be affected. In D3, we will use a **Smooth Away From Expected (SAFE) non-inferiority frontier**, and the non-inferiority margin and significance level will be modified based on the observed confirmed viral rebound risk in the control arm, while preserving power close to 80% and controlling for type 1 error (p<0.03).^39^ This methodology was developed alongside the Statistical Analysis Plan for D3 and maintains interpretability of results if the control event risk differs from assumptions, while accommodating the constraints of a fixed sample size in an already funded trial. The decision to implement the SAFE frontier after the trial was funded, without increasing the sample size, means the type II error is now slightly higher than the conventional 20%, even if the observed risk of viral rebound in the control arm is as originally assumed. This means we may be marginally more likely to conclude DTG/3TC is inferior to control. Future trials should ideally consider an appropriate frontier for the non-inferiority margin at the design stage and plan the sample size accordingly. Notably the use of SAFE in D3 is unbiased, as it was developed and introduced prior to trial data becoming available.

D3 incorporates **a nested PK and safety study** to generate data to support the licensing of the DTG/3TC fixed-dose formulations in children. The inclusion of a nested PK study within a larger trial, as previously done in ODYSSEY,^48,49^ allows completion of the regulatory submissions while the fully powered efficacy trial is still in progress, thus ensuring the new formulations are available by the time the RCT reports its findings.

Modern HIV-1 treatment effectively suppresses viral replication in plasma, but persistence of HIV-1 DNA as integrated proviruses can be responsible for viral rebound at times of reduced ART exposure. In D3, batched genotypic resistance testing will be conducted using **NGS** on **stored buffy coat samples** in addition to traditional Sanger sequencing on saved plasma samples. **NGS-based assays** enable detection of drug resistance, including M184V/I, at very low VLs, addressing concerns regarding the use of 3TC in a 2DR. **The use of b**uffy coat samples provide advantages in handling, processing, stability, storage, and cost savings compared to traditional peripheral blood mononuclear cells. Our validated NGS for resistance testing on buffy coat samples, may pave the way for its more widespread implementation in future research and clinical practice.^40^

### Challenges in study set-up and implementation

D3 was initially designed to compare DTG/3TC to any 3DR used in standard of care. However, given the superiority of DTG-based ART demonstrated in ODYSSEY,^33^ and the changing landscape of paediatric ART, we anticipated DTG-based 3DRs would become the predominant ART used in standard of care during the trial. Allowing the use of non-DTG regimens in the control arm would pose a challenge for interpreting D3 results, with some children receiving knowingly inferior treatments for some of follow-up. Moreover, the anticipated transition to DTG-based 3DR in the control arm during the trial would likely complicate trial comparisons (particularly longitudinal laboratory data influenced by treatments) and reporting. To address these concerns, we amended the protocol before the start of recruitment, to make DTG-based 3DR the control arm. We were unable to provide study drug prior to enrolment, hence children randomised to the control arm who had yet transitioned to DTG were switched to DTG-3DR at enrolment. This deviation from the classic switch design, where patients in the control arm continue their pre-trial treatment, allowed the trial to align with rapidly evolving best treatment practices, and reduced differences between trial arms (as children on non-DTG based ART started DTG at enrolment in both arms).

D3 is a **regulatory trial**, providing data on participants enrolled in the PK and safety substudy for licensing applications of paediatric DTG/3TC formulations before the main trial ends. We introduced various safeguards to preserve the trial’s integrity, with particular support from the DMC whose reviews have been aligned with data provision to the funder.

The trial initially experienced rapid recruitment, primarily enrolling older children in the higher weight bands. We were concerned that this could lead to trial recruitment completing before the PK and safety substudy was fully recruited. To overcome this, **enrolment of children weighing ≥25kg in sub–Saharan Africa and Thailand was capped**, and recruitment for the PK substudy was completed within the intended 12-month timeline.

We were **unable to recruit children in the lowest weight band** (6 to <10kg) due to the absence of virologically suppressed children aged ≥2 years weighing <10kg. While this weight band was included in the trial design to accommodate malnourished and small-for-age children,^50^ it was anticipated that the number would be low since infants often take a long time to achieve virological suppression, even when diagnosed soon after birth.

## Conclusions

D3 is a pioneering trial evaluating DTG/3TC for sustaining virological suppression in children and adolescents in diverse settings. With current ART objectives aimed at enhancing quality of life, the trial will contribute valuable insights into optimising treatment for this population. Through multiple substudies the trial will efficiently evaluate several scientific questions related to DTG-based ART in children. The inclusion of a nested PK and safety substudy will enable licensing approval of DTG/3TC formulations in children by the time the trial ends, showcasing the integration of regulatory studies within large-scale RCTs.

## Abbreviations

2DR: two-drug regimen
3DR: three-drug regimen
3HP: once weekly rifapentine and isoniazid given for three months
3TC: lamivudine
AE: adverse event
ALT: alanine transaminase
ART: antiretroviral therapy
cART: current triple antiretroviral therapy (in the ASPIRE trial)
BIC: bictegravir
CAR: current antiretroviral regimen (in the SALSA trial)
CI: confidence interval
CD4: T-lymphocytes with cluster of differentiation 4 receptor
C-SSRS: Columbia-Suicide Severity Rating Scale
DMC: data monitoring committee
DT: dispersible tablet
DTG: dolutegravir
DTG/3TC: 2-drug regimen with dolutegravir and lamivudine
DNA: deoxyribonucleic acid
EDTA: ethylenediamine tetraacetic acid
eGFR: estimated glomerular filtration rate
EMA: European Medicines Agency
FCT: film-coated tablet
FDA: U.S. Food and Drug Administration
FDC: fixed dose combination
FTC: emtricitabine
HbA1c: haemoglobin A1c
HBV: hepatitis B virus
HBcAb: hepatitis B core antibody
HBsAb: hepatitis B surface antibody
HBsAg: hepatitis B surface antigen
HIV: human Immunodeficiency virus
HRQoL: health related quality of life
INSTI: integrase stand transfer inhibitors
ITT-E: intention-to-treat exposed (population)
LTFU: lost to follow-up
LLQ: lower limit of quantification
MRC CTU at UCL: Medical Research Council Clinical Trials Unit at University College London
NGS: new generation sequencing
NRTI: nucleos(t)ide reverse transcriptase inhibitors
NNRTI: non-nucleoside reverse transcriptase inhibitors
PHPT: The Program for HIV Prevention and Treatment
PK: pharmacokinetic
PD: pharmacodynamic
RNA: ribonucleic acid
RCT: randomised controlled trial
SAE: serious adverse event
SAFE: Smooth Away From Expected
SAP: statistical analysis plan
SOC: standard of care
TAF: tenofovir alafenamide
TB: tuberculosis
TDF: tenofovir disoproxil fumarate
TMG: trial management group
TMT: trial management team
TSC: trial steering committee
ULN: upper limit of normal
VL: viral load
WHO: World health Organization
YTB: youth trial board

## Supplementary Material

Appendix A

Appendix B

## Figures and Tables

**Table 1:** Studies evaluating a two-drug regimen with DTG and 3TC

STUDY	OUTCOME MEASURE	SUPPRESSION/FAILURE RATES AS REPORTED
***Virologically suppressed participants***		
ANRS 167 LAMIDOL: single arm study in adults on first-line ART^4^	Therapeutic success (therapeutic failure included: confirmed VL>50c/mL, interruption of treatment, loss to follow-up, death)	Week 48: 101/104 (97%) (therapeutic failures included 1 confirmed VL>50c/mL, 1 interruption of therapeutic strategy, 1 LTFU by week 48).
ASPIRE: RCT comparing DTG+3TC versus current triple ART (cART) in adults on first-line ART^9^	Treatment failure including confirmed VL≥50c/mL, interruption of treatment, loss to follow-up	Week 24: 3/44 (6.8%) in the DTG+3TC arm (failures included 1 confirmed VL≥50c/mL, 1 treatment discontinuation, 1 LTFU by week 24) versus 3/45 (6.7%) in the cART arm (failures included 2 regimen simplifications, 1 LTFU; treatment difference 0.15%, 90% CI −9.8 to 10.2). FDA snapshot algorithm for VL<50c/mL at 48 weeks: 40/44 (91%) were suppressed in the DTG+3TC arm versus 40/45 (89%) in the cART arm; treatment difference 2.0% (95% CI, −12.6% to 16.5%).
DOLAM: RCT comparing DTG versus DTG+3TC versus SOC in adults on first-line ART^1,8^	Virological failure (VL≥50c/mL)	Week 24 (initial phase): 1/30 (confirmed VL≥50c/mL) in DTG+3TC arm, none in the SOC arm. Week 48 (extended phase): 3/131(2%) in the DTG/3TC arm vs 2/134(1%) in the triple ART arm had confirmed VL≥50c/mL; treatment difference 0-8 (95% CI -3-3 to 5-2).
TANGO: phase III RCT comparing DTG/3TC versus TAF-based 3- or 4-drug ART (TAF-ART)^7,13,14^	Virological failure (VL≥50c/mL) (FDA snapshot; ITT-E)	Week 48: 1/369 (0.3%) in DTG/3TC arm vs 2/372 (0.5%) in TAF-ART arm had VL≥50c/mL; adjusted difference -0.3 (95% CI -1.2 to 0.7). Week 96: 1/369 (0.3%) in DTG/3TC vs 4/372 (1.1%) in TAF- ART had VL≥50c/mL; adjusted difference -0.8 (95% CI -0.2 to 0.4). Week 144: 1/369 (0.3%) in DTG/3TC vs 5/372 (1.3%) in TAF-ART had VL≥50c/mL; adjusted difference -1.1 (95% CI -2.4 to 0.2).
SALSA: phase III RCT comparing DTG/3TC vs current antiretroviral regimen (CAR; 50% NNRTI, 40% INSTI, 10% PI-based)^6^	Virological failure (VL≥50c/mL) (FDA snapshot; ITT-E)	Week 48: 1/246 (0.4%) in DTG/3TC arm vs 3/247 (1.2%) in the CAR arm (adjusted difference, -0.8%; 95% CI, -2.4%, 0.8%) had VL≥50c/mL.
ART-PRO: open-label, single arm in adults receiving DTG/3TC with and without historic 3TC resistance, but no baseline majority 3TC resistance by proviral DNA population (Sanger) genotyping^3,12,15^	Virological suppression <50c/mL (ITT-E FDA snapshot)	Of 41 enrolled, 21 with and 20 without history of 3TC resistance; Remained on DTG/3TC and had VL<50c/mL: Week 48: 38/41 (93%); Week 96: 37/41 (90%); Week 144: 37/41 (90%). Over 144 weeks 4 participants discontinued DTG/3TC prematurely, all with VL<50c/mL at discontinuation; no difference in virological suppression <50c/mL between the groups with and without historic 3TC resistance (18/21 (86%) vs 19/20 (95%), respectively).
SOLAR 3D: prospective (non-randomised) open-label comparative study in adults with prior virologic failure^2,11^	VL ≥50c/mL (FDA snapshot; ITT- E)	Of 100 patients, 50 with and 50 without historic M184V/I. Week 48: 1/50 (2%) with M184V/I and 3/50 (6%) without M184V/I had VL≥50c/mL. Week 96: 2/50 (4%) with M184V/I and 1/50 (2%) without M184V/I had VL≥50c/mL
RUMBA: RCT comparing DTG/3TC vs Bictarvy (BIC/TAF/FTC) in adults on INSTI-based triple ART^10^	Intact replication-competent HIV-1 and total HIV-1 DNA (viral reservoirs) at weeks 48	Week 48: no evidence for a difference in changes in viral reservoirs between baseline and week 48. Study also reported week 48 VL results: 79/81 (97%) in DTG/3TC arm vs 39/49 (98%) in Bictarvy arm had VL <50c/mL.
***ART-naïve participants***		
PADDLE: single arm study in ART-naïve adults^18,21^	FDA snapshot algorithm: proportion with VL<50c/mL (therapeutic failure included VL≥50c/mL, missing VL or interruption of treatment)	Week 48: 18/20 (90%) had VL<50c/mL; therapeutic failures included 1 suicide and 1 participant who had confirmed VL≥50c/mL and discontinued study. Week 96: 18/20 (90%) had VL<50c/mL (no new failures).
ACTG A5353: single arm study in ART-naïve adults^24^	FDA snapshot algorithm: proportion with VL<50c/mL (therapeutic failure included VL≥50c/mL, missing VL or interruption of treatment); Protocol defined virological failure: confirmed VL>400c/mL at week 16 or 20 or confirmed VL>200c/mL at or after week 24	Week 24: 108/120 (90%) had VL<50c/mL; therapeutic failures included 6 participants who had discontinued study/LTFU, 1 with missing VL, 5 with VL≥50c/mL. 3 participants had protocol defined virological failure prior to or at 24 weeks.
GEMINI-1,2: phase III RCT in ART-naïve adults^16,17,19^	Proportion with VL<50 c/mL at weeks 48, 96 and 144 (FDA snapshot; ITT-E)	Week 48 (pooled GEMINI 1 and 2): 655/716 (91%) on DTG+3TC vs 669/717 (93%) on DTG+TDF/FTC had VL<50 c/mL; adjusted difference -1.7% (95% CI -4.4 to 1.1). Week 96 (pooled GEMINI 1 and 2) 616/716 (86%) on DTG+3TC vs 642/717 (90%) on DTG+TDF/FTC had VL<50c/mL; adjusted difference -3.4 (95% CI -6.7 to 0). Week 144 (pooled GEMINI 1 and 2): 584/716 (82%) on DTG+3TC vs 599/717 (84%) on DTG+TDF/FTC had VL<50c/mL; adjusted difference -1.8 (95% CI -5.8 to 2.1).
STAT: single arm ’test and treat’ study in adults started on DTG/3TC within 14 days of HIV diagnosis with unknown VL, CD4, HBV and resistance at baseline^22,23^	Proportion with VL<50 c/ml among all participants (ITT-E missing VL treated as failure); Proportion with VL<50 c/ml of all participants with VL (observed analysis); Proportion with VL<50c/mL and remaining on DTG/3TC of all participants (FDA snapshot)	Through 48 weeks treatment modified in 10 participants (5 HBV co-infection, 1 baseline M184V, 1 rash, 2 participant’s decision, 1 pregnancy). Week 48: 107/131(82%) had VL <50c/mL in ITT-E missing=failure analysis; 107/111(97%) in observed analysis; 100/131 (76%) in FDA snapshot; 26/29 (90%) participants with VL>500,000 c/mL at baseline achieved VL<50 c/mL.
D2ARLING^20^	Proportion with plasma VL <50 c/mL (snapshot algorithm; ITT-E)	Week 24: 100/106 (94%) on DTG/3TC vs 103/108 (95.37%) on DTG+TDF/XTC had VL<50c/mL; difference - 1.03% (-7.9% to 5.8%)
DANCE: single arm study in ART-naïve adolescents aged 12 to <18years^26,27^	Proportion with VL<50 c/ml (ITT-E); Sensitivity analysis performed with participants excluded who could not contribute VL data due to site closure	Week 48: 26/32 (81%) had VL<50c/mL in ITT-E analysis; 26/30 (87%) had VL<50c/mL in sensitivity analysis; Week 96: 22/32 (69%) had VL<50c/mL in ITT-E analysis; 22/25 (88%) in sensitivity analysis.

2DR = two-drug regimens; 3DRs = three-drug regimens; 3TC= lamivudine; ART = antiretroviral therapy; cART = current triple antiretroviral therapy (in the ASPIRE trial); c/mL = copies per millilitre; BIC = bictegravir; CAR = current antiretroviral regimen (in the SALSA trial); DTG/3TC = dolutegravir/lamivudine; FDA = U.S. Food and Drug Administration; FTC = emtricitabine; HBV = hepatitis B virus; INSTI = integrase stand transfer inhibitors; TDF/FTC = tenofovir disoproxil fumarate/emtricitabine; LTFU = lost to follow-up; NNRTI = non-nucleoside reverse transcriptase inhibitors; ITT-E = intention-to-treat exposed (population); RCT = randomised controlled trial; SOC = standard of care; TAF = tenofovir alafenamide; TDF = tenofovir disoproxil fumarate; VL = viral load.

**Table 2:** Participant inclusion and exclusion criteria

Participant inclusion criteria
Children living with HIV-1^a^ who are virologically suppressed for at least the last 6 months prior to enrolment^b,c^ Aged 2 to <15 years old Weight 6 kg or higher Girls who have reached menarche must have a negative pregnancy test at screening and randomisation Girls who are sexually active must be willing to adhere to highly effective methods of contraception^d^ A parent or legal guardian is willing and able to give informed consent on behalf of the child as per national legislation and willing to adhere to the protocol Participant is willing to give informed assent if the trial site clinician deems them old enough and able to understand the age-appropriate information about participation in the study
^a^ Any historic HIV-1 detectable VL or HIV-1 RNA or DNA PCR may be used as confirmation of HIV-1 infection. ^b^ Children will be considered virologically suppressed if they meet either of the following 2 scenarios (A or B) AND any additional VLs between the first VL (used for eligibility) and the screening VL are <50 c/mL: At least two HIV-1 RNA VLs <50 c/mL: a screening VL <50 c/mL and one in >6 to <12 months prior to screening OR At least three HIV-1 RNA VLs <50 c/mL: a screening VL <50 c/mL, one in the ≤6 months prior to screening plus one in the ≥12 to <18 months prior to screening^b^. ^c^ The screening sample VL must be <50 c/mL. For samples prior to screening, a diluted sample may be used; if the VL in the diluted sample is below LLQ, a calculated VL<200 c/mL is allowed; if the VL in the diluted sample is equal or above LLQ the calculated VL should be below 50 c/mL ^d^ Highly effective contraception methods are injectable, implantable, oral and intrauterine contraceptives which have an expected failure rate <1% per year
**Participant exclusion criteria**
Any previous switch in ART regimen for virological, immunological or clinical treatment failure Evidence of previous resistance to lamivudine or INSTI^a^ Any prior use of regimens consisting of single or dual NRTIs with the exception of postnatal antiretroviral prophylaxis (e.g. zidovudine, nevirapine) for prevention of mother to child transmission Known allergy or contraindications to dolutegravir or lamivudine Diagnosis of tuberculosis and on anti-tuberculosis treatment; children can be enrolled after successful tuberculosis treatment^b^ Treatment of co-morbidities with drugs which have significant interactions with antiretroviral treatment, requiring dose adjustment of the study drugs (children can be enrolled after the illness resolves) Randomisation visit more than 12 weeks after the most recent screening visit Positive HBsAg^c^ Screening ALT equal to 3 or more times the upper limit of normal AND bilirubin equal to 2 or more times the upper limit of normal (ALT ≥3xULN AND bilirubin ≥2xULN) Screening ALT equal to 5 or more times the upper limit of normal ALT (≥5xULN) Patients with severe hepatic impairment or unstable liver disease (as defined by the presence of ascites, encephalopathy, coagulopathy, hypoalbuminaemia, oesophageal or gastric varices, or persistent jaundice), or known biliary abnormalities (with the exception of Gilbert’s syndrome or asymptomatic gallstones) Screening creatinine clearance <30 mL/min/1.73m^2 d^ Patients aged ≥6 years at moderate or high risk of suicide as determined by C-SSRS ^e^ Girls who are pregnant or breastfeeding Children who are in the legal custody of the state and do not have a parent or guardian able to provide informed consent on their behalf

a Resistance testing is not required at trial entry.

b Successful tuberculosis treatment is defined as completed anti-tuberculosis treatment, free of tuberculosis-associated symptoms and not known to be smear- or culture-positive in the last month of treatment.

c Screening ALT of more than 2xULN should be further assessed for HBsAg-negative HBV infection and include testing for HBcAb and HBsAb; participants positive for HBcAb and negative for HBsAb should be excluded; participants negative for HBsAg and positive for both HBcAb and HBsAb can be included.

d For calculation of creatinine clearance or estimated glomerular filtration rate in children an updated bedside Schwartz equation should be used eGFR (mL/min/1.73m2)=41.3 x height (m)/serum creatinine (mg/dL)

e See Protocol, Section 5.8.1.C for categorisation of risk of suicide determined by C-SSRS34

ART = antiretroviral therapy; ALT = alanine transaminase; C-SSRS = Columbia-Suicide Severity Rating Scale; eGFR = estimated glomerular filtration rate; HBcAb = hepatitis B core antibody, HBsAb = hepatitis B surface antibody; HBsAg = hepatitis B surface antigen; INSTI = integrase stand transfer inhibitors; LLQ = lower limit of quantification; NRTI = nucleoside reverse transcriptase inhibitors; ULN = upper limit of normal; VL = viral load

**Table 3:** DTG, 3TC and DTG/3TC dosing for children aged 2 to <15 years

WHO weight bands	DTG recommended^a^ once daily dose		3TC recommended^a^ once daily dose	DTG/3TC once daily dose in the D3 trial		DTG dosing in the D3 trial, mg/kg
	5 mg, dispersible tablets	50mg, film-coated tablets		5/30mg, dispersible tablets	50/300mg, film-coated tablets	
**6-<10 kg**	15mg (3 DT)	-	90mg	15/90mg (3 DT)		1.5-2.5 mg/kg (DT)
**10-<14 kg**	20mg (4 DT)	-	120mg	20/120mg (4 DT)	-	1.4-2.0 mg/kg (DT)
**14-<20 kg**	25mg (5 DT)	-	150mg	25/150mg (5 DT)	-	1.3-1.8 mg/kg (DT)
**20-<25 kg**	30mg (6 DT) ^b^	50mg (1 FCT)^b^	180mg or 225mg^c^	30/180mg (6 DT)^d^	50/300mg (1 FCT)^d^	1.2-1.5 mg/kg (DT) 2.0-2.5 mg/kg (FCT)
**>25 kg**	30mg (6 DT) ^b^	50mg (1 FCT)^b^	300mg	-	50/300mg (1 FCT)	≥2.0 mg/kg (FCT)

DT = dispersible tablets; DTG = dolutegravir; FCT = film coated tablets, DTG/3TC = dolutegravir and lamivudine fixed dose combination formulations; 3TC = lamivudine

a Paediatric dolutegravir dosing recommended by World Health Organization (WHO), U.S. Food and Drug Administration (FDA) and European Medicines Agency (EMA)

b Dolutegravir doses 30mg DT and 50mg FCT are equivalent

c For children 20-<25kg WHO-recommended 3TC dose is 180mg, EMA- and FDA-recommended 3TC dose is 225mg

d Children 20-<25kg at the sites where the intensive pharmacokinetic substudy is conducted will be taking DTG/3TC as either six 5/30mg DT or one 50/300mg FCT depending on the formulation allocated to their site for this weight band; participants in non-PK sites will be taking DTG/3TC 50/300mg, unless they are not able to swallow tablets.

**Table 4:** Primary and secondary outcome measures in the D3 trial

Primary outcome measure
Proportion of children with confirmed viral rebound (defined as the first of two consecutive HIV-1 RNA ≥50 c/mL) by week 96
**Secondary efficacy outcome measures^a^**
Proportion of children with confirmed viral rebound (defined as the first of two consecutive HIV-1 RNA ≥50 c/mL) by week 48 Proportion of children with confirmed HIV-1 RNA ≥50c/mL at weeks 48 and 96 (modified FDA snapshot)^b^ Proportion of children with HIV-1 RNA ≥50c/mL at weeks 24, 48 and 96 (including blips and confirmed measures ≥50c/mL) New resistance-associated mutations in those with confirmed HIV-1 RNA ≥50c/mL Time to any new or recurrent WHO 3 or WHO 4 event or death Change in CD4 (absolute count and percentage) from baseline to weeks 24, 48 and 96
**Secondary safety outcome measures^a^**
Incidence of serious adverse events, grade ≥3 clinical and laboratory adverse events Incidence of adverse events leading to discontinuation or modification of the treatment regimen Proportion of children with a change in ART for toxicity or switch to second-line ART Change in blood lipids from baseline to weeks 48 and 96 Change in creatinine clearance estimated using bedside-Schwartz formula to weeks 48 and 96
**Secondary patient-reported outcome measures^a^**
Assessed by participant/caregiver completed questionnaires: Adherence to antiretroviral treatment Acceptability of antiretroviral treatment Sleep and mood assessment Suicidal ideation and behaviour Health-related quality of life

a Outcomes are compared through trial follow-up unless otherwise specified.

b The standard FDA snapshot algorithm classifies deaths and adverse events related to toxicity which lead to treatment change (where no previous VL in window) as “missing virological data”. In D3, we will use a modified FDA snapshot algorithm and classify these events by last VL (“rebound” if VL≥50 c/mL or “missing virological data” if <50 c/mL).

**Table 5:** Choice of non-inferiority margin and significance level based on observed confirmed viral rebound risk using the Smooth Away From Expected (SAFE) frontier.

Observed confirmed viral rebound risk (P0)^a^	1%	2%	3%	4%	5%	6%	7%	8%		9%	10%	11%	12%	13%	14%	15%
**Non-inferiority Margin**	5.0%	5.8%	6.5%	7.3%	8.0%	8.9%	9.5%	9.9%		10.0%	10.0%	10.0%	**10.0%**	10.0%	10.0%	10.0%
**Significance level**	0.50%	0.50%	0.50%	0.50%	0.50%	0.50%	0.50%	0.50%		2.50%	2.50%	2.50%	**2.50%**	2.50%	2.50%	2.50%


**Power**	91.8%	83.8%	79.0%	75.9%	74.2%	74.1%	74.9%	75.9%		77.2%	78.6%	79.3%	**78.8%**	77.3%	75.2%	73.0%
**Type 1 error**	3.06%	2.86%	2.69%	2.72%	2.77%	2.65%	2.62%	2.52%		2.52%	2.58%	2.58%	**2.58%**	2.57%	2.56%	2.56%
**P (change margin)^b^**	100%	100%	100%	99.9%	98.8%	95.0%	86.0%	71.4%		53.6%	36.2%	22.0%	**12.1%**	6.1%	2.8%	1.2%

The column in bold corresponds to the sample size calculation assumption made at the initial design stage.

a The choice of non-inferiority margin and significance level will depend on the observed confirmed viral rebound risk. The power, type 1 error and probability of changing margin depend on the true control event risk.

b The probability of changing the margin is the probability that, for a given true control event risk, the observed control event risk will be lower than 9%, hence leading to using a non-inferiority margin in the analysis different from the originally planned 10%.

## Data Availability

The D3 trial data are held at MRC CTU at UCL, which encourages optimal use of data by employing a controlled access approach to data sharing (http://www.ctu.mrc.ac.uk/our_research/datasharing/), incorporating a transparent and robust system to review requests and provide secure data access consistent with the relevant ethics committee approvals. We will consider all requests for data sharing, which can be initiated by contacting the corresponding author or through the URL: https://www.ctu.mrc.ac.uk/our-research/other-research-policy/data-sharing/application-process/

## References

[1] Blanco JL, Rojas J, Paredes R (2018). Dolutegravir-based maintenance monotherapy versus dual therapy with lamivudine: a planned 24 week analysis of the DOLAM randomized clinical trial. The Journal of antimicrobial chemotherapy.

[2] Blick G, Cerreta E, Mancini G, Cosenza A (2021). SOLAR 3D: A Prospective Study Switching to DTG/3TC from 3-or 4-Drug ART for Maintenance of Viral Suppression with Historic M184V/I Mutation and Prior Virological Failures: 48 Week Primary Endpoint Results.

[3] De Miguel Buckley R, Rial-Crestelo D, Montejano R (2022). Long-term Evaluation of Residual Viremia in a Clinical Trial of Dolutegravir Plus Lamivudine as Maintenance Treatment for Participants With and Without Prior Lamivudine Resistance. Open Forum Infect Dis.

[4] Joly V, Burdet C, Landman R (2019). Dolutegravir and lamivudine maintenance therapy in HIV-1 virologically suppressed patients: results of the ANRS 167 trial (LAMIDOL). The Journal of antimicrobial chemotherapy.

[5] Li JZ, Sax PE, Marconi VC (2019). No Significant Changes to Residual Viremia After Switch to Dolutegravir and Lamivudine in a Randomized Trial. Open Forum Infect Dis.

[6] Llibre JM, Brites C, Cheng CY (2023). Efficacy and Safety of Switching to the 2-Drug Regimen Dolutegravir/Lamivudine Versus Continuing a 3- or 4-Drug Regimen for Maintaining Virologic Suppression in Adults Living With Human Immunodeficiency Virus 1 (HIV-1): Week 48 Results From the Phase 3, Noninferiority SALSA Randomized Trial. Clin Infect Dis.

[7] Osiyemi O, De Wit S, Ajana F (2022). Efficacy and Safety of Switching to Dolutegravir/Lamivudine Versus Continuing a Tenofovir Alafenamide-Based 3- or 4-Drug Regimen for Maintenance of Virologic Suppression in Adults Living With Human Immunodeficiency Virus Type 1: Results Through Week 144 From the Phase 3, Noninferiority TANGO Randomized Trial. Clin Infect Dis.

[8] Rojas J, de Lazzari E, Negredo E (2021). Efficacy and safety of switching to dolutegravir plus lamivudine versus continuing triple antiretroviral therapy in virologically suppressed adults with HIV at 48 weeks (DOLAM): a randomised non-inferiority trial. Lancet HIV.

[9] Taiwo BO, Marconi VC, Berzins B (2018). Dolutegravir Plus Lamivudine Maintains Human Immunodeficiency Virus-1 Suppression Through Week 48 in a Pilot Randomized Trial. Clinical Infectious Diseases.

[10] Blomme E, Trypsteen W, Delporte M (2022). Abstract M042 HIV Drug Therapy Glasgow 2022.

[11] Blick G, Cerreta E, Mancini G, Cosenza A, Fang L Prior M184V/I and multiple prior virological failures have no impact on the efficacy of switching HIV+ adults to DTG/3TC through 96Wks in SOLAR-3D. Abstract OAB0202.

[12] Rial-Crestelo D, de Miguel R, Montejano R (2021). Long-term efficacy of dolutegravir plus lamivudine for maintenance of HIV viral suppression in adults with and without historical resistance to lamivudine: Week 96 results of ART-PRO pilot study. The Journal of antimicrobial chemotherapy.

[13] van Wyk J, Ajana F, Bisshop F (2020). Switching to DTG/3TC fixed-dose combination (FDC) is non-inferior to continuing a TAF-based regimen (TBR) in maintaining virologic suppression through 96 Weeks (TANGO Study. Abstract O441 HIV Drug Therapy Glasgow.

[14] van Wyk J, Ajana F, Bisshop F (2020). Efficacy and Safety of Switching to Dolutegravir/Lamivudine Fixed-Dose 2-Drug Regimen vs Continuing a Tenofovir Alafenamide-Based 3- or 4-Drug Regimen for Maintenance of Virologic Suppression in Adults Living With Human Immunodeficiency Virus Type 1: Phase 3, Randomized, Noninferiority TANGO Study. Clin Infect Dis.

[15] De Miguel R, Rial-Crestelo D, Dominguez-Dominguez L (2020). Dolutegravir plus lamivudine for maintenance of HIV viral suppression in adults with and without historical resistance to lamivudine: 48-week results of a non-randomized, pilot clinical trial (ART-PRO). EBioMedicine.

[16] Cahn P, Madero JS, Arribas JR (2019). Dolutegravir plus lamivudine versus dolutegravir plus tenofovir disoproxil fumarate and emtricitabine in antiretroviral-naive adults with HIV-1 infection (GEMINI-1 and GEMINI-2): week 48 results from two multicentre, double-blind, randomised, non-inferiority, phase 3 trials. Lancet.

[17] Cahn P, Madero JS, Arribas JR (2020). Durable Efficacy of Dolutegravir Plus Lamivudine in Antiretroviral Treatment-Naive Adults With HIV-1 Infection: 96-Week Results From the GEMINI-1 and GEMINI-2 Randomized Clinical Trials. Journal of acquired immune deficiency syndromes (1999).

[18] Cahn P, Rolon MJ, Figueroa MI, Gun A, Patterson P, Sued O (2017). Dolutegravir-lamivudine as initial therapy in HIV-1 infected, ARV-naive patients, 48-week results of the PADDLE (Pilot Antiretroviral Design with Dolutegravir LamivudinE) study. J Int AIDS Soc.

[19] Cahn P, Madero Sierra, Arribas JR (2022). Three-year durable efficacy of dolutegravir plus lamivudine in antiretroviral therapy - naive adults with HIV-1 infection. Aids.

[20] Cordova E, Hernandez Rendon J, Mingrone V Efficacy of dolutegravir plus lamivudine in treatment-naive people living with HIV without baseline drug-resistance testing: week 24 results of the randomized D2ARLING study. Abstract TUPEB02.

[21] Figueroa MI, Rolón MJ, Patterson P, Gun A, Cahn P, Sued O Dolutegravir-Lamivudine as initial therapy in HIV-1 infected, ARV-naïve patients: 96 week results of the PADDLE trial Abstract MOPEB0287.

[22] Rolle CP, Berhe M, Singh T Feasibility, efficacy, and safety of dolutegravir/lamivudine (DTG/3TC) as a first-line regimen in a test-and-treat setting for newly diagnosed people living with HIV (PLWH): 48-week results of the STAT study.

[23] Rolle CP, Berhe M, Singh T (2021). Dolutegravir/lamivudine as a first-line regimen in a test- and-treat setting for newly diagnosed people living with HIV. Aids.

[24] Taiwo BO, Zheng L, Stefanescu A (2018). ACTG A5353: A Pilot Study of Dolutegravir Plus Lamivudine for Initial Treatment of Human Immunodeficiency Virus-1 (HIV-1)-infected Participants With HIV-1 RNA <500000 Copies/mL. Clin Infect Dis.

[25] Radford M, Parks DC, Ferrante S, Punekar Y (2019). Comparative efficacy and safety and dolutegravir and lamivudine in treatment naive HIV patients. Aids.

[26] Puthanakit T, Aurpibul L, Lopez M Efficacy and Safety of the 2-Drug Regimen Dolutegravir/Lamivudine (DTG/3TC) in Antiretroviral Therapy (ART)-Naive Adolescents Living With HIV-1: DANCE Study Week 48 Results.

[27] Puthanakit T, Aurpibul L, Lopez M Efficacy and Safety of Dolutegravir/Lamivudine (DTG/3TC) in Antiretroviral Therapy (ART)-Naive Adolescents Living With HIV-1: DANCE Study Week 96 Results. Abstract EPB0250.

[28] Stanford University (2022). HIV Drug Resistance Database. NRTI Resistance Notes.

[29] Kabra M, Barber T, Allavena C (2022). Abstract P081 HIV Drug Therapy Glasgow 2022.

[30] Evitt LA, Kumar R, Kamath R (2021). Effectiveness and Tolerability of DTG + 3TC in Clinical Practice: Evidence in PLHIV from Real-world Data. Abstract 898 IDWeek 2021.

[31] Letang E, Priest J, di Giambenedettoa S Effectiveness and tolerability of the 2-drug regimen dolutegravir plus lamivudine in people with HIV-1: a systematic literature review of real-world evidence from clinical practice.

[32] Foster C, Ayers S, Fidler S (2020). Antiretroviral adherence for adolescents growing up with HIV: understanding real life, drug delivery and forgiveness. Ther Adv Infect Dis.

[33] Turkova A, White E, Mujuru HA (2021). Dolutegravir as First- or Second-Line Treatment for HIV-1 Infection in Children. N Engl J Med.

[34] The Columbia Lighthouse Project The Columbia-Suicide Severity Rating Scale (C-SSRS).

[35] European AIDS Clinical Society Panel and Penta HIV Guidelines Working Group (2021). EACS/Penta Guidelines Version 11.

[36] World Health Organization (2021). Consolidated guidelines on HIV prevention, testing, treatment, service delivery and monitoring: recommendations for a public health approach.

[37] Lewis LL, Venzon D, Church J (1996). Lamivudine in children with human immunodeficiency virus infection: a phase I/II study. The National Cancer Institute Pediatric Branch-Human Immunodeficiency Virus Working Group. The Journal of infectious diseases.

[38] Turkova A, Moore CL, Butler K (2018). Weekends-off efavirenz-based antiretroviral therapy in HIV-infected children, adolescents and young adults (BREATHER): Extended follow-up results of a randomised, open-label, non-inferiority trial. PLoS One.

[39] Quartagno M, Chan M, Turkova A, Ford D, White IR (2023). The Smooth Away From Expected (SAFE) non-inferiority frontier: theory and implementation with an application to the D3 trial. Trials.

[40] Botha JC, Byott M, Spyer MJ (2023). Sensitive HIV-1 DNA Pol Next-Generation Sequencing for the Characterisation of Archived Antiretroviral Drug Resistance. Viruses.

[41] World Health Organization (2021). Call to accelerate the study of new drugs for HIV in pregnant and breastfeeding women.

[42] U.S. Department of Health and Human Services, National Institutes of Health, National Institute of Allergy and Infectious Diseases, Division of AIDS (2017). Division of AIDS (DAIDS) Table for Grading the Severity of Adult and Pediatric Adverse Events, Corrected Version 2.1.

[43] NIH Division of Microbiology and Infectious Diseases (DMID) Pediatric toxicity tables (2007). https://www.niaid.nih.gov/sites/default/files/dmidpedtox.pdf.

[44] World Health Organization (2010). Antiretroviral therapy for HIV infection in infants and children: towards universal access. Recommendations for a public health approach - 2010 revision.

[45] Medical Dictionary for Regulatory Activities (MedDRA) https://www.meddra.org/.

[46] Morris TP, Walker AS, Williamson EJ, White IR (2022). Planning a method for covariate adjustment in individually randomised trials: a practical guide. Trials.

[47] Moore CL, Turkova A, Mujuru H (2021). ODYSSEY clinical trial design: a randomised global study to evaluate the efficacy and safety of dolutegravir-based antiretroviral therapy in HIV-positive children, with nested pharmacokinetic sub-studies to evaluate pragmatic WHO-weight-band based dolutegravir dosing. BMC Infect Dis.

[48] Bollen P, Turkova A, Mujuru H (2018). CROI.

[49] Waalewijn H, Bollen P, Moore C Pharmacokinetics of dolutegravir 5mg dispersible tablets in children weighing 6 to <20kg dosed using WHO weight bands.

[50] Sterling TR, Scott NA, Miro JM (2016). Three months of weekly rifapentine and isoniazid for treatment of Mycobacterium tuberculosis infection in HIV-coinfected persons. Aids.

