# Supplementary material for: D3/Penta 21 clinical trial design: a randomised non-inferiority trial with nested drug licensing substudy to assess dolutegravir and lamivudine fixed dose formulations for the maintenance of virological suppression in children with HIV-1 infection, aged 2 to 15 years
Source: Contemp Clin Trials. Author manuscript; Available in PMC 2025 Sep 23. (PMC7618164; doi:10.1016/j.cct.2024.107540)
Supplement: Appendix A [file EMS207920-supplement-Appendix_A.pdf]

Table of Contents

D3 Trial Team 2

Figure S1. Choice of non-inferiority margin based on observed confirmed viral rebound risk using the Smooth Away From Expected (SAFE) frontier 4

Table S1. Trial asseessment schedule 5

Questionnaires overview 6

Figure S2. D3 Adherence Questionnaire (Parent/Carer version) 7

Figure S3. D3 Medicines Acceptability Questionnaire (Parent/Carer version) 8

Figure S4. D3 Mood Questionnaire (Parent/Carer version) 13

Figure S5. D3 Sleep Questionnaire (Parent/Carer version) 14

Modified FDA Snapshot Algorithm 15

Table S2. Modified FDA snapshot algorithm .... **Error! Bookmark not defined.**

D3 Substudies 17

References 18

D3 Trial Team

Fondazione Penta ETS, Padova, Italy: Carlo Giaquinto, Alessandra Nardone, Gabija Morkunaite
Penta – Child Health Research, Padova, Italy: Carlo Giaquinto
MRC CTU at UCL: Anna Turkova, Debbie Ford, Man Chan, Gabriela Toledo, Elizabeth James, Mags Thomason, Nazia Parkar, Iona White, Anna Parker, Anas Omar, Zainab Alkurwi, Moira Spyer, Katja Doerholt, Stephen Townsend, Hannah Sweeney, Margaret Hook, Lee Barker, Lu Gao, Matteo Quartagno, Diana Gibb
AMS – PHPT CTU: Tim Cressey, Suwalai Chalermpantmetagul, Rukchanok Peongjakta, Chutipa Meeboon, Worathip Sripaoraya, Warunee Khamjakkaew, Namthip Kruenual, Pra-ornsuda Sukrakanchana, Ampika Kaewbundit
Baylor College of Medicine Children's Foundation, Uganda: Adeodata Kekitiinwa, Pauline Amuge, Christine Namugwanya, Lameck Kiyimba, Resty Babirye Okello, Florence Namuli, Ronald Nabimba, Angella Baita, Dickson Bbuye, Susan Tukamuhebwa, Rachael Namuddu Kikabi, Rose Jacqueline Kadhuba, Henry Balwa, Sarah Nabukalu, Muzamil Nsibuka Kisekka, Anthony Kirabira, Lekku Lawrence, Judith Tikabibamu, Maria Benita Aino, Gerald Agaba Muzorah, Collins Mujyanama, Annet Nalugo, Priscilla Namubiru
Joint Clinical Research Centre, Uganda: Cissy Kityo, Victor Musiime, Elizabeth Kaudha, Annet Nanduudu, Caroline Otike, Emmanuel Mujyambere, Dridah Nakiboneka, Barbara Mukanza, Julius Tumusiime, Onen Gilbert, Ritah Mbabazi, Abigail Atwine, Priscilla Kyobutungi, Juliet Ategeka, Alex Musiime, Sharif Musumba, Rashidah Nazzinda, Nicholas Wangwe, Phyllis Mwesigwa, Diana Rutebarika, Jameena Elsauko, Mangadalen Nansaigi, Mercy Tukamushaba, Alice Mulindwa, Aidah Nakalyango, Ocitti Paul, Christine Nambi, Milly Ndigendawani, Mariam Naabalamba, Eram David, Odochi Denis, Baliruno David, Ezra Lutalo, Eddie Rubanga, Josephine Namusanje, Josephine Kobusingye, Disan Mulima, Maria Nannungi, Faith Balmoi, Charles Draleku, Faith Mbasani, Crispus Katemba, Juliet Ankunda, Julian Tusiime
Makerere University – John Hopkins University Research Collaboration, Uganda: Philippa Musoke, Grace Miriam Ahimbisibwe, Hajira Kataike, Winnie Nansamba, David Balamusani, Rosemary Namwanje, Enoch Mulwanyi, Gerald Bright Businge, Maxensia Owor, Aziida Nabukeera, Ruth Nakku, Zainab Nakivumbi Nassoma, Immaculate Nayiga Serunjogi, Barbara Musoke Nakirya, Sarah Nakabuye, Erinah Kyomukama, Rebecca Wampamba, Joseph Ouma, Stella Nalusiba, Emmanuel Mayanja, Donald Wagaana, Zaam Zinda Nakawungu, Sarah Babirye Ssebabi, Olivia Higiro Kaboggoza, Edith Nabawubye, Mildred Kabasonga, Harriet Namusisi, Judith Nampewo, Agnes Mary Mugagga, Richard Isabirye, Francis Sserugo, Annet Kawuma, Agnes Namuddu, Joanita Nankya Baddokwaya, Juliet Nanyonjo, Winifred Kaahwa, Maria Musisi, Paula Mubiru Namayanja, Doreen Twenatwine, Robert Byuma, Winifred Luwedde, Margaret Mugenyi, Joseph Mutebo, Francis Katongole, Fabian Okello, Max Kiwewa, Ronald Okwera, Derick Balungi, Obed Tumwizere, Teopista Nakyanzi, Ann Kankindi, Maria Janine Nambusi, Ivan Rukundo, Henry Odyek, Barnabas Weere, Bosco Kafufu, David Ssebunya, Africano Kamugisha, Emmanual Hakizimana, Charles Nyende, Evelyn Akurut, Johnson Tumwesigye, Brenda Catherine Kakayi, Rebecca Sakwa, Mark Ssenyonga, Joseph Semakula, Joyce Mwebaza, Judith Kainza, Miscah Babirye Otim
Durban International Clinical Research Site, Enhancing Care Foundation, South Africa: Moherndran Archary, Rosie Mngqibisa, Tiyara Arumugam, Sundrapragasen Pillay, Raziya Bobat, Nombuso Nkosi, Nozibusiso Rejoice Mosia, Sheleika Singh, Shingirai Chimene, Jabu Mkhulise, Thandokuhle Mncube, Innocentia Thandokuhle Mncube, Happiness Magwaza, Noncebo Siphesihle Gumede, Zethu Mnyandu
Perinatal HIV Research Unit, Matlosana, South Africa: Ebrahim Variava, Tumelo Moloantoa, Nadia Sabet, Ryan Sabet, Modiehi Mosala, Itumeleng Holele, Eva Mogotsi, Angelinah Montwedi, Serame Mokoena, Keabetswe Kotsokoane, Dineo Rampai, Avy Violari, Lerato Maretlwa, Palesa Tshipunyane, Iris Matotong, Abraham Mammwn Pattamukkil, Nkazimulo Xulu, Sthembiso Mhlanga, Ofhani Harmious Makhari, Gifty Okyere Manu, Zukisa Mpeluza, Tumelo Moloantoa, Mbusiseni Ngema, Abdul Kaka, Rieta Stokes, Nadia Bellingan, Linique Le Grange, Leoni Stytler, Zakkiyya Jeeva

Perinatal HIV Research Unit, Soweto, South Africa: Avy Violari, Afaaf Liberty, Mandisa Nyati, Haseena Cassim, Lindiwe Maseko, Dipuo Dhlomoza, Precious Ndebele, Jackie Brown, Emily Lebotsa, Deirdre Josipovic, Mantwa Kunene, Sisinyana Ruth Mathiba, Tryphinah Madonsela, Nasreen Abrahams, Zaakirah Essack, Shamelle Govender, Tshepo Magoje, Amanda Tawana, Zandisile Mtshali, Thabile Degracia Hlomuka, Valerie Khemese
Chiangrai Prachanukroh Hospital, Thailand: Pradthana Ounchanum, Suchada Ruenglerdpong, Areerat Kongponoi, Kanyanee Kaewmamueng, Warunee Srisuk, Yupawan Thaweesombat, Sukanda Denjanta, Jutarat Thewsoongnoen, Naowarat Kunyanone
Kalasin Hospital, Thailand: Sakulrat Srirojana, Doungjai Donngern, Petcharat Phunkhum, Arisara Kamkoonmongkol, Thananya Naksomboon, Nonthaporn Na Kalasin
Khon Kaen Hospital, Thailand: Ussanee Srirompotong, Athiporn Rungsapphaiboon, Wallapa Daechasatain, Wanchalerm Boonsub, Patamawadee Sudsaard, Thunyasiri Dechboran, Manthana Mitchai, Thanawat Samranphit, Orapin Wannasri, Kriangkrai Kongsuk
Nakornping Hospital, Thailand: Suparat Kanjanavanit, Thannapat Chankun, Chayakorn Saewtrakool, Pacharaporn Yingyong, Raungwit Junkaew, Benjawan Thomyota
Prapokklao Hospital, Thailand: Kanokkorn Sawasdichai, Chaiwat Ngampiyaskul, Nantika Paiboon, Benjama I-nala, Wanna Chamjamrat, Pisut Greetanukroh, Chanthaporn Imbumroong, Sasipass Khannak, Rattana Chahmeanprabnakorn
Birmingham Heartlands Hospital, UK: Steven Welch, Melanie Rooney, Laura Thrasyvoulou, Sue Fagg, Katie Price, Baldip Kaur, Molly Williams
Great Ormond Street Hospital, UK: Anna Turkova, Alasdair Bamford, Delane Shingadia, Jade Sugars, Olamide Alimi, Sophie Santry, Carolyn Chan, Kelly Cripps
St Mary's Hospital, UK: Caroline Foster, Isabel Johnson, Katy Bridges
Hospital Sant Joan de Déu, Spain: Clàudia Fortuny, Antoni Noguera-Julian, Cristina López Hidalgo, Sílvia Caudras Ferrando, Alba Murciano, Miriam Coto, Kenia Sánchez, Bonaventura Ruiz
Hospital Universitario 12 de Octubre, Spain: Pablo Rojo, Cristina Epalza, Luis Prieto, Jose Tomas Ramos, Irene Hernández Perez, Lilit Manukyan, Manuel Gijon
PK substudies: David Burger, Angela Colbers, Tom Jacobs, Lisanne Bevers
Pharmacogenomics substudy: Dan Carr, Andrew Owen, Rebecca Jensen
Virology substudy: Eleni Nastouli, Moira Spyer, Matt Byott, Ian Botha
Health Economics substudy: Paul Revill, Simon Walker
Youth Trials Board: Magda Conway, Lungile Jafta, Mercy Shibemba
Trial Steering Committee Members: Hermione Lyall, Elizabeth Maleche Obimbo, Theodore Ruel, Alex Compagnucci, Imelda Mahaka, Fanele Bulose, Gugulethu Bomela, Anna Turkova, Cissy Kityo, Tim Cressey, Avy Violari
Data Monitoring Committee Members: Anton Pozniak, Jane Crawley, Rodolphe Thiébaut, Helen McIlleron (for consideration of PK substudy)

Figure S1. Choice of non-inferiority margin based on observed confirmed viral rebound risk using the Smooth Away From Expected (SAFE) frontier

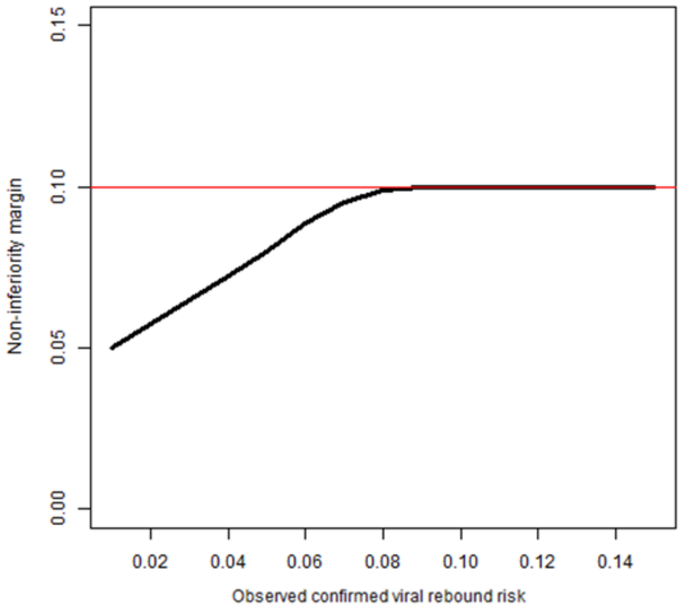

Table S1. Trial asseessment schedule

Study week number	Screen W-12 to W0	W0	W4	W12	W24	W36	W48	W60	W72	W84	W96	Further follow-up	End of study visit
Signed Informed consent	X	Confirm											
Clinical assessment [1]	X	X	X	X	X	X	X	X	X	X	X	Every 12 wks	X
Dispensing antiretroviral drugs		X	X	X	X	X	X	X	X	X	X	Every 12 wks	X
**Laboratory Assessments**													
Pregnancy test (urine) [2]	X	X	X	X	X	X	X	X	X	X	X	Every 12 wks	X
HBsAg	X												X
HIV-1 RNA VL [4]	X	(X)	(X)	(X)	X	(X)	X	(X)	(X)	(X)	X	At least every 48 wks	X
Haematology [5]	X	X*	X	X	X	(X)	X	(X)	(X)	(X)	X	As per local practice	X
Biochemistry [6]	X	X*	X	X	X	(X)	X	(X)	(X)	(X)	X	As per local practice	X
Lipase (same draw as biochemistry)		X	X	X	X								
Lipids (same draw as biochemistry)		X					X				X	As per local practice	(X)
Lactate		X	X	X	X								
HbA1c		X			X		X		X		X	As per local practice	(X)
T-cell lymphocyte subsets (same draw as haematology) [11]		X	(X)	(X)	X	(X)	X	(X)	X	(X)	X	At least every 24 wks	X
Urine dipstick [12]		X					X				X	Every 48 wks	X
**Other assessments**													
Adherence assessment [13]		X	X	X	X	X	X	X	X	X	X	Every 12 wks	X
Acceptability questionnaire [14]		X	X		X		X		X		X	Every 24 wks	X
Sleep and mood questionnaires [14]		X	X		X		X		X		X	Every 24 wks	X
C-SSRS questionnaire (children aged ≥6 years)	X	X	X	X	X	X	X	X	X	X	X	Every 12 wks	X
HRQoL questionnaire/assessment		X	X		X		X		X		X	Every 24 wks	X
**Storage of the samples**													
Plasma storage [15]		X	X	X	X	X	X	X	X	X	X	Every 12 wks	X
Buffy coat storage (same draw as plasma storage) [16]		X	X	X	X	X	X	X	X	X	X	Every 12 wks	X
Sparse PK samples storage [17]			X		X		X		X		X		
Pharmacogenomics [17]		X											

Children enrolled at in South Africa and Uganda randomised to DTG/3TC and weighing <40kg were asked for additional consent to participate in the Intensive Pharmacokinetic and Safety substudy (see below). Every effort is made to minimise loss to follow up. Children who miss clinic visits are traced using home visits and mobile phone calls. If a participant or their carer chooses to discontinue participation in the trial, they are asked to allow their data from routine care to be collected to inform the study outcomes (see Protocol, section 6.7). At withdrawal or loss to follow-up the trial clinician is asked to rate the participant's health (good, reasonable, poor, very poor), providing information on any association between withdrawal/loss to follow-up and poor health outcomes. C-SSRS = Columbia-Suicide Severity Rating Scale; HbA1AC = haemoglobin A1c ; HBsAg = hepatitis B surface antigen; HRQoL = health related quality of life; PK = pharmacokinetic; VL=viral load.

( ) Optional if done in routine care.

[1] Clinical assessment includes medical and ART history, clinical examination, weight, height, mid upper arm circumference, waist circumference, paediatric WHO staging for HIV and adverse events (starting from week 0).

[2] For girls who have reached menarche.

[4] Real-time/local VL to be done at screening, weeks 24, 48 and 96 and then every 48 weeks (with confirmatory VLs for HIV-1 RNA ≥50 c/mL); more frequent VLs may be done if site routine VLs are more frequent. An additional VL is required if treatment failure is suspected. Retrospective VL testing is performed using routine stored plasma [15] at the scheduled trial visits when a real-time VL is not done.

[5] Haematology: haemoglobin, red blood cells, mean corpuscular volume, white blood cells, lymphocytes, neutrophils, platelets.

[6] Biochemistry: urea, creatinine, albumin, alanine transaminase, aspartate transaminase, alkaline phosphatase, bilirubin.

[11] CD3+, CD4+, CD8+ T-lymphocyte percentage and absolute, total lymphocyte count.

[12] Urine dipstick for proteinuria.

[13] Pill count (except week 0) and adherence questionnaire

[15] At all scheduled trial visits plasma samples are stored for future batch testing for retrospective VL, and low-level viraemia and resistance testing in a subset of samples. Additionally, a plasma sample is stored at unscheduled visits if treatment failure is suspected (all trial participants).

[16] At all scheduled trial visits, buffy coat samples are stored (from the same draw as plasma storage) [15]. The stored samples are used for the evaluation of the total HIV-1 DNA and resistance mutations on HIV-1 proviral DNA using the next generation sequencing (NGS). Additional buffy coat from EDTA blood sample is stored at unscheduled visits if treatment failure is suspected (all trial participants) and at DTG/3TC discontinuation prior to 48-week follow-up for reasons other than confirmed viral rebound (participants consented for the Intensive PK substudy).

[17] For sites in South Africa, Thailand and Uganda

Questionnaires overview

The D3 trial utilises participant/carer questionnaires to evaluate participant adherence to treatment and acceptability of study medicines, mood and sleep, suicidal ideation and behaviour and health-related quality of life. All questionnaires have been translated to local languages used at the trial sites.

The adherence questionnaires were adapted from questionnaires used in previous paediatric trials conducted by MRC Clinical Trial Unit (CTU) at UCL/Penta (BREATHER NCT01641016, ODYSSEY NCT02259127) and capture: missed doses over the last week and the last month; reasons for not taking medications as prescribed (adapted from Vreeman R, et al.[1]); and self-assessed or carer-assessed adherence using a 5-point Likert scale (excellent, very good, fair, not that good, poor).

The Medicine Acceptability questionnaires were adapted for children from Scott S, et al.[2,3] and capture: the ways the medicines are taken; participant and carer's views on how easy it is to take the study medicines and on how well the medicines work and whether they make the child ill.

Mood questionnaires were based on subscales of the validated Revised Children's Anxiety and Depression Scale;[4] to identify symptoms of generalised anxiety and low mood (Additional File 2, p 8). Sleep questionnaires were adapted from the Pittsburgh questionnaire,[5] focusing on the key questions relevant to children, including time taken to fall asleep, frequency of waking up during the night, frequency of experiencing bad or vivid dreams and frequency of "trouble staying awake at school or during everyday activities". These adapted Sleep questionnaires were first used in the ODYSSEY trial.[6]

As dolutegravir is associated with suicidality ideation and behaviour, this is evaluated in the trial using the validated Columbia-Suicide Severity Rating Scale scale[7] in children aged 6 years and older. The risk of suicide is assessed as mild, moderate or severe based on the responses and the guidance on risk-based management is provided at the end of the questionnaire. Children were ineligible for the trial if they had a moderate or high risk of suicide at screening or enrolment but remain in trial follow-up if a risk is identified post enrolment.

Health-related quality of life is evaluated by a validated EQ-5D-Y questionnaire.[8]

Figure S2. D3 Adherence Questionnaire (Parent/Carer version)

D3

Participant Number: ☐☐☐☐☐☐☐☐☐

3 Letter Code: ☐☐☐

Date of Birth: d d / m m m / y y y y

Adherence Questionnaire Parent/Carer
Version 2.0 05 Jan 2022

Visit week:	0 ☐	4 ☐	12 ☐	24 ☐	36 ☐	48 ☐	60 ☐	72 ☐	84 ☐	96 ☐	108 ☐	120 ☐
	132 ☐	144 ☐	156 ☐	168 ☐	180 ☐	Unscheduled ☐						

Visit date: d d / m m m / y y y y

THE FOLLOWING INFORMATION SHOULD BE ENTERED INTO THE 'Adherence Questionnaire Parent/Carer' eCRF

TO BE COMPLETED BY THE CLINICAL TEAM

Completed by Carer Alone? Yes ☐ No ☐ *If No who else was involved?* ____________

TO BE COMPLETED BY THE PARENT/CARER

1. What is your relationship to the child? Parent/Carer ☐ Other ☐ **1a.** If Other, specify:____________

2. How does your child take their antiretroviral medicine(s) *(Tick all that apply)*

☐ My child takes their medicine(s) independently.
☐ I remind my child or support them to take their medicine(s) (but they take them on their own).
☐ I give my child their medicine(s) or supervise them to take them.

We know that it is difficult taking medication every day. Most people miss doses from time to time and it is rare that people take medication perfectly. We are interested in finding out what it is like for you and your child.

3. Has your child missed any doses in the last week? Yes ☐ No ☐

4. Has your child missed any doses in the last month? *(excluding the last week)* Yes ☐ No ☐ I don't know ☐

5. There are many reasons why people do not take their medication. Did your child miss their medication for any of these reasons? *(Tick all that apply)*

☐ a. My child didn't miss any *(Go to Q6)*
☐ b. My child had run out of medicine(s)
☐ c. My child forgot
☐ d. The timing of the medicine(s) is difficult
☐ e. My child did not have any food to take their medicine(s) with
☐ f. My child's routine was different from normal (e.g. holidays, sleepovers etc.)
☐ g. I was not always around to give the medicine(s)
☐ h. My child did not want other people to know they were taking medicine(s)
☐ i. My child doesn't like the taste of the medicine(s)
☐ j. My child was unwell or was vomiting
☐ k. My child had low mood or was feeling too sad
☐ l. My child refused to take the medicine(s)
☐ m. My child was fed up or tired of taking the medicine(s)
☐ n. My child thinks the medicine(s) is harmful
☐ o. My child has doubts about taking medicine(s) due to their beliefs
☐ p. My child had difficulty swallowing the medicine(s)
☐ q. Other *(Complete Q5a)*

5a. Please specify____________________

6. How would you rate your child's adherence (taking antiretroviral medication every day as prescribed) since the last visit? *(Please tick the statement you think is most true to you)*

Excellent ☐ Very good ☐ Fair ☐ Not that good ☐ Poor ☐

7. Thank you for taking the time to fill out this form. Please add any comments you have:

____

____

FOR CLINICAL USE:

Questionnaire administered by (Name):______________________________________

(Signature): ______________________________ Date of completion: d d / m m m / y y y y

Figure S3. D3 Medicines Acceptability Questionnaire (Parent/Carer version)

D3

Participant Number: ☐☐☐☐☐☐☐☐☐

3 Letter Code: ☐☐☐

Date of Birth: d d / m m m / y y y y

Medicines Acceptability Questionnaire - Parent/Carer

Version 4.0 05 Jan 2022

Visit	0 ☐	4 ☐	24 ☐	48 ☐	72 ☐	96 ☐	120 ☐	144 ☐	168 ☐	Unscheduled ☐
Visit date:	d d / m m m / y y y y									

THE FOLLOWING INFORMATION SHOULD BE ENTERED INTO THE 'MAQ Parent/Carer eCRF

TO BE COMPLETED BY CLINICAL TEAM

Completed by Parent/Carer alone? Yes ☐ No ☐ If No who else was involved? ____________________

TO BE COMPLETED BY THE PARENT/CARER

1. What is your relationship to the child? Parent/Carer ☐ Other ☐ **1a.** If Other, specify____________________

2. What type of antiretroviral medication does your child take?

(If your child takes more than one type of formulation please tick all applicable boxes)

☐ Film coated tablet(s) (tablets are hard and don't disperse/break down easily in water)

☐ Dispersible tablet(s) (tablets disperse/break down easily in water)

☐ Syrup

☐ Other

2a. Please specify 'Other'____________________

3. Who prepares your child's medicine(s)? (*Tick one box)*

☐ I/Parent/Carer prepare(s) my child's medicine(s)

☐ My child prepares their medicine(s) independently

☐ Not applicable — medicine(s) don't need preparation (i.e. unbroken tablets are taken direct to mouth)

4. How easy is it give the medicine to your child?

☐ My child takes the medicine(s) **easily by themselves.**

☐ My child takes the medicine(s) **easily, but with help.**

☐ My child takes the medicine(s) **with help, but requires persuasion.**

☐ My child **is made to take the medicine(s)** against their will.

MAQ Parent/Carer—v4.0 05 Jan 2022 Page 1 of 5

D3

Participant Number: ☐☐☐☐☐☐☐☐☐

3 Letter Code: ☐☐☐

Date of Birth: d d / m m m / y y y y

5. How does your child take their **film coated tablets**? *(If your child does not take film coated tablets, continue to Q6)*

5a. How does your child take their film coated tablet(s)?

☐ Direct to mouth

☐ Mixed in liquid or food

5b. Do you or your child cut/crush your film coated tablet(s) to make them easier to take?

☐ Yes

☐ No

5c. Are the tablets taken with any liquid?

☐ No

Yes, with:

☐ Water

☐ Other

5d. Please specify 'Other'__________________

This section is for participants who take <u>dispersible tablets</u>. If your child does not take dispersible tablets, please continue to Q11.

6. How does your child take their **dispersible tablet(s)**? *(If your child does not take dispersible tablets continue to Q11)*

6a. How does your child take their dispersible tablet(s)?

☐ Dispersed in liquid

☐ Direct to mouth

6b. For taking their dispersible tablet(s) does your child use?

☐ Dosing cup (the cup that came with your child's medicine)

☐ Syringe

☐ Other

6c. Please specify 'Other' ____________________

6d. Into what liquid are the tablet(s) dispersed?

☐ None

☐ Water

☐ Other

6e. Please specify 'Other'__________________

7. Do you think the dosing cup or syringe are helpful for preparing and giving your child the medicine(s)? For each of the following statements, please tick **ONE** box that best describes what you think.

**I think ...**	Very Unhelpful	Unhelpful	Neither Helpful nor Unhelpful	Helpful	Very Helpful	N/A, I do not use this item
**7a.** The dosing cup is.........	☐	☐	☐	☐	☐	☐
**7b.** The syringe is........	☐	☐	☐	☐	☐	☐

8. If your child takes <u>dispersible tablet(s)</u> with a dosing cup or syringe how easy is it for you to clean them? For each of the following statements, please tick **ONE** box that best describes what you think.

**I think ...**	Very Hard	Hard	Neither Easy nor Hard	Easy	Very Easy	N/A, I do not use this item
**8a.** Cleaning the dosing cup is.........	☐	☐	☐	☐	☐	☐
**8b.** Cleaning the syringe is........	☐	☐	☐	☐	☐	☐

D3

Participant Number: ☐☐☐☐☐☐☐☐☐

3 Letter Code: ☐☐☐

Date of Birth: d d m m m y y y y

9. After your child has taken their medicine(s), how often do you add more water to the dosing cup, swill, and have your child drink again?

- ☐ Always
- ☐ Sometimes
- ☐ Not at all/rarely
- ☐ N/A, I do not use the dosing cup

10. How much of the medicine(s) does your child normally swallow?

- ☐ All of it
- ☐ Most of it
- ☐ Very little of it

11. How **easy** is it for your child to take this medicine(s)? For each of the following statements, please tick **ONE** box that best describes what your child thinks.

**My child thinks…**	Very Hard	Hard	Neither Easy nor Hard	Easy	Very Easy
**11a.** The number of times they have to take the medicine(s) each day is ………… *(e.g. once a day or twice a day)*	☐	☐	☐	☐	☐
**11b.** The amount of medicine(s) they have to take is………… *(e.g. number of tablets or volume of liquid)*	☐	☐	☐	☐	☐
**11c.** Taking medicine(s) when they are not at home is ………… *(consider the need for water or ease of carry-*	☐	☐	☐	☐	☐

12. How does this medicine(s) **look, smell and taste to your child?** For each of the following statements, please tick **ONE** box that best describes your opinion.

**My child thinks …**	Very Bad	Bad	Neither Good nor Bad	Good	Very Good
**12a.** The medicine(s) tastes …………	☐	☐	☐	☐	☐
**12b.** The aftertaste is ………… *(consider the taste that stays in the mouth after you swallow the medicine(s))*	☐	☐	☐	☐	☐
**12c.** The texture of the medicine(s) is ………… *(e.g. smooth or fizzy)*	☐	☐	☐	☐	☐
**12d.** The colour of the medicine(s) is …………	☐	☐	☐	☐	☐
**12e.** The smell of the medicine(s) is …………	☐	☐	☐	☐	☐

Participant Number: ☐☐☐☐☐☐☐☐☐

3 Letter Code: ☐☐☐

Date of Birth: d d / m m m / y y y y

13. How easy is it for your child to swallow the medicine(s)? For the following statement, please tick ONE box that best describes your opinion.					
**My child thinks...**	Very Hard	Hard	Neither Easy nor Hard	Easy	Very Easy
**13a.** Swallowing the medicine(s) is	☐	☐	☐	☐	☐

**14.** What does your child think of the **packaging** of the medicine(s)? For each of the following statements, please tick ONE box that best describes your opinion.					
**My child thinks...**	Very Bad	Bad	Neither Good nor Bad	Good	Very Good
**14a.** The packaging of the medicine(s) is............	☐	☐	☐	☐	☐

15. It is also important to know how <u>you</u> feel about the medicine(s). Therefore, if you are the person responsible for preparing and/or administering the medicine(s), please tell us how you think the medicine(s) **looks, smells and handles**? For each of the following statements, please tick **ONE** box that best describes your opinion.

Please answer not applicable if your child prepares and administers their medicine(s) independently or you personally do not prepare or administer the medicine(s).

**I find ...**	Very Hard	Hard	Neither Easy nor Hard	Easy	Very Easy	N/A
**15a.** Seeing the medicine(s) is............	☐	☐	☐	☐	☐	☐
**15b.** Holding the medicine(s) is	☐	☐	☐	☐	☐	☐
**I think ...**	Very Bad	Bad	Neither Good nor Bad	Good	Very Good	N/A
**15c.** The packaging of the medicine(s) is............	☐	☐	☐	☐	☐	☐

**16.** How does this medicine(s) **work?** For each of the following statements, please tick **ONE** box that best describes your opinion.					
**The medicine(s) my child takes...**	Definitely not	Probably not	Possibly works	Probably works	Definitely works
**16a.** Works and does what is supposed to do *(e.g. helps their symptoms or prevents further illness).*	☐	☐	☐	☐	☐

D3

Participant Number: ☐☐☐☐☐☐☐☐☐

3 Letter Code: ☐☐☐

Date of Birth: d d / m m m / y y y y

17. Does this medicine(s) give your child side effects or make them feel ill? For each of the following statements, please tick ONE box that best describes your opinion.

**The medicine(s) my child takes...**	Never	Seldom	Sometimes	Often	Always
**17a.** Makes them feel ill	☐	☐	☐	☐	☐

17b. If your child feels ill after taking their medicine(s), for how long do they feel ill? For each of the following statements, please tick **ONE** box that best describes your opinion.

(If you ticked 'never' to Q17a, please skip Q17c and continue to Q18)

**After they take their medicine(s)....**	For less than an hour	Between 1-3 hours	For half of the day	For more than half of the day	All day
**17c.** My child feels ill..........	☐	☐	☐	☐	☐

18. Please tell us overall how **happy** you think **<u>your child</u>** is with their medicine(s). Please **CROSS** the number that best describes how they feel. (⊗)

Very unhappy — Very happy

0 — 1 — 2 — 3 — 4 — 5 — 6 — 7 — 8 — 9 — 10

19. Please tell us overall how **happy <u>you</u>** are with your child's medicine(s). Please **CROSS** the number that best describes how you feel. (⊗)

Very unhappy — Very happy

0 — 1 — 2 — 3 — 4 — 5 — 6 — 7 — 8 — 9 — 10

20. Finally, please use the space below to describe **you and your child's experiences** using this medicine(s) which this questionnaire has not covered. We are interested in all comments whether they are positive (how easy the medicine is to give/take) or negative (any difficulties you or your child experience).

FOR CLINICAL USE:

Questionnaire administered by (Name):______________________________

(Signature): ____________________ **Date of completion:** d d / m m m / y y y y

Figure S4. D3 Mood Questionnaire (Parent/Carer version)

D3

Participant Number: ☐☐☐☐☐☐☐☐☐

3 Letter Code: ☐☐☐

Date of Birth: d d / m m m / y y y y

Mood Questionnaire Parent/Carer

Version 2.0 04 Jan 2022

Visit	0 ☐	4 ☐	24 ☐	48 ☐	72 ☐	96 ☐	120 ☐	144 ☐	168 ☐	Unscheduled ☐

Visit date: d d / m m m / y y y y

THE FOLLOWING INFORMATION SHOULD BE ENTERED INTO THE 'Mood Questionnaire Parent/Carer' eCRF

TO BE COMPLETED BY THE CLINICAL TEAM

1. Completed by Parent/Carer alone? Yes ☐ No ☐ Q1a. If No who else was involved?__________________

TO BE COMPLETED BY THE PARENT/CARER

Please put a circle around the ONE word that shows how often each of these things happen to your child.

There are no right or wrong answers.

**2.**	**0**	**1**	**2**	**3**
a. My child worries about things	Never	Sometimes	Often	Always
b. My child worries that something awful will happen to some-one in the family	Never	Sometimes	Often	Always
c. My child worries that bad things will happen to him/her	Never	Sometimes	Often	Always
d. My child worries that something bad will happen to him/her	Never	Sometimes	Often	Always
e. My child worries about what is going to happen	Never	Sometimes	Often	Always
f. My child thinks about death	Never	Sometimes	Often	Always
**3.**	**0**	**1**	**2**	**3**
a. My child feels sad or empty	Never	Sometimes	Often	Always
b. Nothing is much fun for my child anymore	Never	Sometimes	Often	Always
c. My child has trouble sleeping	Never	Sometimes	Often	Always
d. My child has problems with his/her appetite	Never	Sometimes	Often	Always
e. My child has no energy for things	Never	Sometimes	Often	Always
f. My child is tired a lot	Never	Sometimes	Often	Always
g. My child cannot think clearly	Never	Sometimes	Often	Always
h. My child feels bad about themselves	Never	Sometimes	Often	Always
i. My child feels like he/she doesn't want to move	Never	Sometimes	Often	Always
j. My child feels restless	Never	Sometimes	Often	Always

FOR CLINICAL USE:

Questionnaire administered by (Name):____

(Signature): ______________________________ **Date of completion:** d d / m m m / y y y y

Mood Questionnaire Parent/Carer—v2.0 04 Jan 2022 Page 1 of 1

Figure S5. D3 Sleep Questionnaire (Parent/Carer version)

D3

Participant Number: ☐☐☐☐☐☐☐☐☐

3 Letter Code: ☐☐☐

Date of Birth: d d / m m m / y y y y

Sleep Questionnaire Parent/Carer
Version 2.0 04 Jan 2022

Visit week:	0 ☐	4 ☐	24 ☐	48 ☐	72 ☐	96 ☐	120 ☐	144 ☐	168 ☐	Unscheduled ☐

Visit date: d d / m m m / y y y y

THE FOLLOWING INFORMATION SHOULD BE ENTERED INTO THE 'Sleep Questionnaire Parent/Carer' eCRF

SLEEP WITHIN THE LAST MONTH

1. In the last month, what time did your child usually go to bed at night? *(Please answer using the 24 hour clock)*

☐☐ : ☐☐

2. In the last month, how long did your child usually take to fall asleep each night? *(Please tick one answer only)*

Less than 15 minutes ☐ 15 minutes to half an hour ☐ Half an hour to an hour ☐ More than an hour ☐

3. In the last month, approximately how many hours of sleep did your child get each night? ☐

(Please write the number of hours)

In the last month...<br>*(Please tick only one box per question)*	Not during the past month	Less than once a week	Once or twice a week	Three or more times a week
**4. How often did it take longer than 30 minutes for your child to fall asleep?**				
**5. How often did your child wake during the night?**				
**6. How often did your child experience bad or vivid dreams ?**				
**7. How often has your child had trouble staying awake at school or during everyday activities?**				

8. In the last month, how would you rate your child's sleep quality overall? *(Please tick one answer only)*

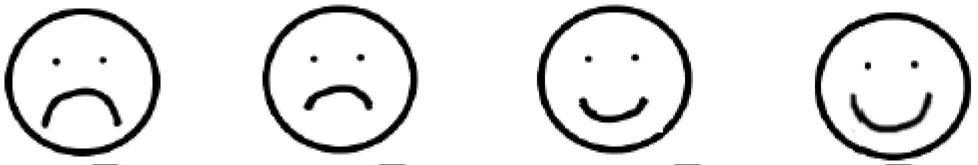

Very Bad ☐ Fairly Bad ☐ Fairly Good ☐ Good ☐

THANK YOU FOR TAKING THE TIME TO FILL OUT THIS FORM

FOR CLINICAL USE:

Questionnaire administered by (Name):______________________________

(Signature): ______________________ Date of completion: d d / m m m / y y y y

Sleep Questionnaire Parent/Carer—v2.0 04 Jan 2022 Page 1 of 1

Modified FDA Snapshot Algorithm

This appendix outlines the modified version of the FDA snapshot algorithm, which will be used to compare virological rebound (HIV-1 RNA ≥50c/mL) in DTG/3TC vs control at weeks 48 and 96.

PERMITTED ART CHANGES

Permitted ART changes include changes of ART components that are unrelated to treatment failure, toxicity, pregnancy, or protocol deviation or patient/carer decision (resulting in ≥7 days off allocated regimen). These have been divided into those that will be (i) ignored and (ii) permitted.

Ignored ART changes in both arms include changes where all components of the ART regimen remain unchanged:

- Dose increases/decreases of the same ART for weight change
- Dose change of 3TC for creatinine clearance
- Move to double DTG dose for TB treatment and return to single dose following completion of TB treatment or similar dose adjustments due to drug-drug interactions with other concomitant medications
- Switch to fixed dose ART combination (FDC) (or FDC to single/dual) containing the same ART components
- Changes between mornings and evenings
- Changes between branded and generic products

Permitted changes include changes where one or more component of the ART regimen has been changed, but the reason for change is not related to toxicity, failure, pregnancy, or protocol deviation or parent/carer decision (resulting in ≥7 days off allocated regimen).

Permitted ART changes in the DTG/3TC arm include:

- Change of ART component(s) for protocol deviation or patient/carer decision provided that the participant switches back to their allocated regimen <7 days after change.

Permitted ART changes in the control arm include:

- Change of ART component(s) due to treatment simplification
- Change of ART component(s) due to child's growth
- Change of ART component(s) due to change in country guidelines or stock-out at clinic
- Change of ART component(s) due to protocol deviation or patient/carer decision provided that the participant switches back to their allocated regimen <7 days after change.

Examples of permitted changes during the trial in the control arm include:

- Change of ABC or ZDV to TDF for children reaching eligible age or weight cut off for TDF
- Change of ABC, ZDV or TDF to TAF if TAF-containing formulations become available for corresponding age/weight bands

NON-PERMITTED ART CHANGES

Non-permitted changes in both arms include:

- Change of ART component(s) due to lack of efficacy or adverse event
- Change of ART component(s) due to pregnancy or desire to become pregnant
- Change of ART component(s) due to protocol deviation or patient/carer decision where the time off the allocated regimen is ≥7 days
- Change of ART components due to any other reasons not listed as ignored or permitted ART changes.

Categorisation of Virological Outcomes at 48/96 weeks (within window 42-54/90-102 weeks)
A modified version of the FDA snapshot algorithm will be used to describe virological failure at weeks 48 and 96 as described in the table below.

Modified FDA snapshot algorithm

	DTG/3TC	SOC
	N (%)	N (%)
**HIV RNA≥50 c/mL[1]**		
**Treatment difference (95% CI)**		
**HIV RNA<50 c/mL[2]**		
**No virological data in week 48/96 window**		
Discontinued study regimen due to AE or death and last on treatment HIV-1 RNA <50 c/mL[3]		
Discontinued study regimen for other reasons and last on treatment HIV-1 RNA <50 c/mL[4]		
On study regimen[5] but missing HIV RNA data in window		

[1]Includes (i) participants on study drug (DTG/3TC) or SOC (including with prior permitted changes while HIV-RNA<50 c/mL) who had confirmed HIV-RNA≥50 c/mL in 48/96 week window; (ii) participants who changed any component of initial regimen because of lack of efficacy prior to/during week 48/96 window; (iii) participants who discontinued/changed any component of initial regimen for reasons other than lack of efficacy prior to/during week 48/96 with the last on treatment (prior to/on the date of change) HIV-1 RNA ≥50 c/mL
[2] Includes (i) participants on study drug (DTG/3TC) or SOC (including with prior permitted changes while HIV-RNA<50 c/mL) and HIV-RNA<50 c/mL at week 48/96
[3]Includes participants who discontinued any component of initial regimen for toxicity/death before or during week 48/96 where last on treatment HIV-RNA<50 c/mL
[4]Includes participants who discontinued or changed any component of initial regimen for reasons other than an AE/death or lack of efficacy, e.g., withdrew consent, lost to follow-up, pregnancy (or desire to become pregnant), transferred care to a non-study site or had non-permitted change of any component of initial regimen before or during week 48/96 where last on treatment HIV-RNA<50 c/mL
[5]DTG/3TC or SOC (including with prior permitted changes while HIV-RNA<50 c/mL)

D3 Substudies

An Intensive Pharmacokinetic (PK) and safery substudy is nested in the trial to support regulatory approvals of DTG/3TC formulations for children. The substudy is conducted in the intervention arm only and aims to evaluate the pharmacokinetics, safety, tolerability and antiviral activity of DTG/3TC in virologically suppressed children aged ≥2 years and weighing <40kg using WHO weight band-aligned dosing. The substudy planned to recruit ≥8 children with evaluable PK curves per WHO weight-band and formulations (Table 3); and ≥14 children per age group 2 to <6 years and ≥6 years. Seven plasma samples were taken at least 7 days after starting DTG/3TC regimen (and ≥21 days after starting DTG) over 24 hours (t=0 (prior to observed dosing), 1, 2, 3, 4, 6 and 24h post-dosing). The substudy fully recruited in approximately 12 months.
The substudy fully recruited in approximately 12 months. PK analyses are being conducted at the department of Pharmacy of the Radboud University Medical Center in Nijmegen, the Netherlands.

Exploratory Population Pharmacokinetic, Pharmacogenetic and Pharmacodynamic analyses will be conducted in all participants at the sites in South Africa, Thailand and Uganda. Participants, whose parents or legal guardians gave consent, had a sample stored for pharmacogenomics at baseline and samples are stored for PK modelling of dolutegravir and lamivudine at weeks 4, 24, 48, 72 and 96. Genomic DNA extraction from whole blood and genetic analysis will be undertaken at the department of Molecular and Clinical Pharmacology, University of Liverpool, using a genome-wide association study and global single nucleotide polymorphism array which will include genes encoding enzymes previously implicated in metabolism and disposition of DTG and/or 3TC. Analysis will be undertaken to explore associations of variant alleles with variability in PK-related outcomes as well as efficacy and toxicity. Any significantly associated variants will be incorporated into PK/PD modelling analyses, performed by Radboud University Medical Center, Nijmegen.

A Tuberculosis-Pharmacokinetic (TB-PK) substudy will assess the pharmacokinetics of DTG and 3TC in HIV/TB co-infected children receiving twice-daily dolutegravir and rifampicin-based TB treatment to substantiate the findings from the ODYSSEY TB-PK substudy.[9] Participants in both arms at the PK sites in South Africa and Uganda who develop TB while in the trial will be invited to join the substudy with an additional consent. The design of the sub-study is reported elsewhere.[9]

A 3HP-DTG PK substudy will explore co-administration of once weekly rifapentine and isoniazid for three months (3HP) for TB prevention with DTG-based two-drug therapy (DTG/3TC) and DTG-based 3DR. The substudy will compare the impact of 3HP on DTG plasma concentrations in children receiving once-daily and twice-daily DTG, using a cross-over design, with children being their own controls. Participants who are likely to benefit from TB prevention therapy as assessed by their treating clinician will be invited to join the substudy with an additional consent after they complete their 96-week visit in the main trial. The PK analysis will be done at the Radboud University Medical Center, Nijmegen.

A Virology substudy will compare very low level viraemia at 1-5 c/mL, and total HIV-1 DNA, as a surrogate measure of HIV-1 reservoir size,[10] between the trial arms. Plasma and buffy coats will be collected at all scheduled visits from enrolment according to the trial assessment schedule. The analyses will be done at the University College London Hospitals Advanced Pathogen Diagnostics Unit.

A Health Economics substudy will evaluate net health benefits and cost-effectiveness of DTG/3TC compared to DTG-based 3DR. If DTG/3TC is shown to be non-inferior to 3DR, it is likely that there will be substantial cost savings associated with taking one less drug for the lifetime of an HIV-infected person. The substudy is led by Centre for Health Economics, University of York.

References

1. Vreeman RC, Nyandiko WM, Liu H, Tu W, Scanlon ML, Slaven JE, et al. Comprehensive evaluation of caregiver-reported antiretroviral therapy adherence for HIV-infected children. AIDS and behavior. 2015;19(4):626-34.
2. The Medication Acceptability Questionnaire (MAQ). https://www.uea.ac.uk/pharmacy/research/maq Accessed 18 Aug 2023.
3. Scott S, Clark A, May H, Bhattacharya D. Validation and Feasibility of the Medication Acceptability Questionnaire to Investigate Tablet and Liquid Alendronic Acid with Older Hospital Patients. Pharmacy (Basel). 2018;6(3).
4. The Child Outcome Research Consortium (CORC). Revised Children's Anxiety and Depression Scale (and Subscales) (RCADS). https://www.corc.uk.net/outcome-experience-measures/revised-childrens-anxiety-and-depression-scale-rcads/ Accessed 18 Aug 2023.
5. Buysse DJ, Reynolds CF, 3rd, Monk TH, Berman SR, Kupfer DJ. The Pittsburgh Sleep Quality Index: a new instrument for psychiatric practice and research. Psychiatry Res. 1989;28(2):193-213.
6. Turkova A, White E, Kekitiinwa AR, Mumbiro V, Kaudha E, Liberty A, et al. Neuropsychiatric manifestations and sleep disturbances with dolutegravir-based antiretroviral therapy versus standard of care in children and adolescents: a secondary analysis of the ODYSSEY trial. Lancet Child Adolesc Health. 2023.
7. The Columbia Lighthouse Project. The Columbia-Suicide Severity Rating Scale (C-SSRS). https://cssrs.columbia.edu/the-columbia-scale-c-ssrs/researchers/ Accessed 18 Aug 2023.
8. The EuroQol Group. The EQ-5D-Y (Youth) questionnaire. https://euroqol.org/eq-5d-instruments/eq-5d-y-about/ Accessed 18 August 2023.
9. Turkova A, Waalewijn H, Chan M, Bollen P, Bwakura-Dangarembizi M, Kekitiinwa A, et al. Dolutegravir twice-daily dosing in children with HIV-associated tuberculosis: a pharmacokinetic and safety study within the open-label, multicentre, randomised, non-inferiority ODYSSEY trial. Lancet HIV 2022;9:e627-e637.
10. Tagarro A, Chan M, Zangari P, Ferns B, Foster C, De Rossi A, et al. Early and Highly Suppressive Antiretroviral Therapy Are Main Factors Associated With Low Viral Reservoir in European Perinatally HIV-Infected Children. Journal of acquired immune deficiency syndromes (1999). 2018;79(2):269-76.
