# Supplementary material for: D3/Penta 21 clinical trial design: a randomised non-inferiority trial with nested drug licensing substudy to assess dolutegravir and lamivudine fixed dose formulations for the maintenance of virological suppression in children with HIV-1 infection, aged 2 to 15 years
Source: Contemp Clin Trials. Author manuscript; Available in PMC 2025 Sep 23. (PMC7618164; doi:10.1016/j.cct.2024.107540)
Supplement: Appendix B [file EMS207920-supplement-Appendix_B.pdf]

To be presented on local headed paper

To be presented on local headed paper

[Remove text box and insert relevant logos here]

Version 3.0 Date 04-October-2021

D3 Study

We are inviting your child to take part in a research study called D3

- Please take time to read the following information carefully. Discuss it with friends and relatives if you wish. Take time to decide whether or not you wish for your child to take part.
- You are free to decide whether or not your child takes part in this research study. If you choose for them to not take part, this will not affect the care your child gets from their doctors in any way.
- Your child can stop taking part in the study at any time, without giving a reason.
- Ask us if there is anything that is not clear or if you would like more information.
- Thank you for reading this information. If you decide for your child to take part you will be given a copy of this information sheet and asked to sign a consent form. You will get a copy of that as well.

Important things that you need to know

- We want to find out whether taking two anti-HIV medicines has fewer side effects and works as well as taking three medicines, in children and young people.
- We are testing a combination of two anti-HIV medicines called dolutegravir (DTG) and lamivudine (also called 3TC). We call these "DTG/3TC". Many children and young people already take three anti-HIV medicines where one of them is DTG, this combination will be used as the control and will be compared to DTG/3TC.
- All participants in the study will be divided into two different treatment groups (DTG/3TC group and control group).
- If your child is allocated to the DTG/3TC group, they will receive the new combination of medicines.
- If your child is allocated to the control group and they are already taking DTG plus two-anti-HIV medicines, they will continue with the same medicines. If they are not already taking DTG plus two anti-HIV medicines, they will switch to receive this combination.
- DTG has been shown to be effective, with few side effects, and 3TC also has few side effects and has been used in children for a long time. The most commonly reported side effects of these medicines are headache, feeling sick and diarrhoea. Your child may already be taking one or both of these medicines as part of their current treatment with the combination of three or four anti-HIV medicines.
- This study will require your child to visit the clinic every three months. There will be an extra visit near the beginning of the study at four weeks after your child joins. Your travel expenses will be reimbursed.

Contents

1 Why are we doing this study?
2 Why is my child being asked to take part?
3 What do I need to know about the medicines used in this study?
4 What will my child need to do if they take part?
5 What are the possible side-effects?
6 What are the possible benefits of taking part in this study?
7 What are the possible disadvantages and risks of taking part?
8 More information about taking part
9 Contacts for further information

1 Why are we doing this study?

This study is for the treatment of HIV.

What is HIV?

HIV is a virus that attacks the cells that help the body fight infection. This means that a person living with HIV may be more likely to become ill as they are not able to fight off other infections and cope with other illnesses.

If you want to know more about HIV, talk to the doctor or nurse who is treating your child.

How is HIV usually treated?

HIV is usually treated by taking three or four different medicines. Some of these medicines can be combined into one tablet. Depending on the medicines used, tablets may need to be taken either one or two times every day.

What are we trying to find out?

There is a new tablet made up of two different medicines, dolutegravir (DTG) and lamivudine (3TC). This tablet is already being used to treat adults with HIV.

In adults, the DTG/3TC combination works really well. Adults only have to take it once a day and they do not have many side effects. However, it is possible that for children, the new medicine won't work as well as the other treatments currently available, which is why it is important to do this study. If it does work well, this could be an excellent medicine for children and young people with HIV.

The **D3 study** will see how children and young people taking **DTG/3TC** are doing compared to others that take DTG plus two other anti-HIV medicines.

2 Why is my child being asked to take part?

The D3 study is happening in five countries: South Africa, Spain, Thailand, Uganda and the United Kingdom. We need a total of 370 children and young people from these countries to take part.

Your child is being asked because:

- They have only ever taken one type of HIV treatment or they never had treatment which didn't work against the virus.
- The virus in their blood has been very low or undetectable each time it has been checked in the last year.

3 What do I need to know about the medicines used in this study?

DTG and 3TC are two medicines that are already used to treat HIV in children, young people and adults. DTG has been shown to work very well when it is taken with two other anti-HIV medicines and is well-tolerated, meaning that people who take it do not have many side effects. 3TC is a medicine that has been used for a long time to treat HIV in combination with other medicines. It has also been shown not to cause many side effects. Both of these medicines only need to be taken once per day.

The medicines used in the DTG/3TC group are tablets which combine these two anti-HIV treatments.

The medicines used in the control group are standard anti-HIV treatments that are already used to treat children and young people with HIV.

4 What will my child need to do if they take part?

Can my child definitely take part?

Not everyone will be able to take part in this study. We need to do some tests first to see whether your child is able to take part. Before we do these tests we will go through this information sheet together. We will give you a copy to keep. You will be asked to sign a consent form to show that you have understood the information and have agreed for your child to take part. If your child is aware of their HIV status and is able to understand the study we will also ask them to give assent. They will then need to have some blood tests and a check-up where the study doctor will examine your child, take a medical history and certain measurements, such as height and weight. It usually takes a few weeks to get the results from these blood tests. Your child's study doctor or nurse will be able to tell you how long it is likely to take to get the results back at your clinic. The total amount of blood taken at this visit will be around three teaspoons. If your child has started having their periods, then a pregnancy test will also be done and, if the pregnancy test is positive, your child will not be able to join the study.

What if the tests show my child can take part?

If the tests show that your child can take part, we will invite them to join the D3 study. We will ask you to confirm that you have fully understood the study and that you still give your consent for your child to join.

There will be two different treatment groups in the D3 study. These are:

- **Group 1 ('DTG/3TC'):** your child will take the new combination of two anti-HIV medicines, DTG and 3TC.
- **Group 2 ('Control'):** your child will take DTG plus two anti-HIV medicines that are already given to children and young people.

Everyone who takes part will be in one of these two groups.

Which group will my child be in?

It is important that the groups receiving each treatment are as similar as possible at the start of the study. To ensure that this happens, a process called randomisation is used to allocate people to each group. This means that a computer program will choose which treatment your child will take. Neither your child, you, nor your child's doctor will be able to choose which group your child is in.

What will happen to my child during the study?

1. If the test results are okay and you still want your child to join the D3 study, your child will then need to attend for another visit. Certain measurements, such as your child's height and weight, will be taken again, and if your child could become pregnant, we will do another pregnancy test. If the result is positive then they will not be able to take part in the study. We will also do some more blood tests at this visit.
2. A computer programme will then decide which treatment group your child will be in – either the new anti-HIV combination, DTG/3TC, or the control treatment of DTG and two other anti-HIV medicines. The study team will tell you and your child about the medicines that your child will be taking and how they should take them.
3. A nurse and doctor will see your child four weeks and 12 weeks after they start taking part in the D3 study, and then every 12 weeks (three months) after that. This is similar to the number of visits that most children and young people living with HIV would have even if they were not taking part in the study. The nurse will talk to you and your child about any problems that your child has been having. The doctor will examine your child and look carefully for any new problems. We will give you new supplies of your child's anti-HIV medicines and any other medicines that they need. We will take a small amount of blood from your child at each visit and will always make sure that the total amount of blood taken is safe for your child's weight.
4. Children and young people who are among the first starting the study and who are allocated to the DTG/3TC group may be invited to take part in a special study to look at the levels of DTG and 3TC in their blood – these are called "pharmacokinetic (PK) studies". This will depend on which clinic your child is treated at. If your clinic is running this PK substudy, your doctor may talk to you and your child about taking part and you will be given another information sheet with more details on this.
5. Your child will be in the study for two to three and a half years. We will watch their health closely during this time and, if for any reason the study doctor has any concerns at these visits, they will change your child's treatment in the normal way, even if they are in the DTG/3TC group. You should let your doctor know of any other medicines that your child is taking in case they

could affect or be affected by the study treatment. You should always ask the study staff for advice before your child starts taking any other medicines.

6. When the study ends, if your child is doing well they may be able to remain on the same anti-HIV medicines they have been taking during the study, but you will need to discuss this with their doctor.

What checks and tests will be done?

At the beginning of the study (during the first six months), all children and young people in both groups will have blood tests done at every visit to check for any side effects and to see if your child is doing well on the medicines. After six months in the study, the blood tests may not be as often and will be similar to how often the blood tests are done routinely as standard care at your clinic.

In addition to the blood tests that your child usually has in clinic, we will collect and store an additional blood sample at each visit. Part of this sample will be stored to check the amount of virus in the blood, using commonly available tests as well as new research tests, and the rest will be stored and used to look at how the body fights HIV.

We will use part of the blood sample collected at some visits to test how much of the medicine that your child is taking is actually getting into their blood, where it can fight the HIV virus. At the first study visit, we would also like to collect and save a small blood sample in order to do genetic testing (see below).

We will ask your child to provide a urine sample at the start of the trial and then approximately every year and, if your child has started having their periods, we will also do a pregnancy test at every trial visit.

Over the course of the study, we will ask you and your child to complete questionnaires asking how your child takes their medicines, what it is like taking medicine and how they feel in general, as we are interested in finding out what children, young people and their carers feel about the study and how it is affecting their wellbeing.

Storing blood samples for future use

After the tests are done for this study there may be some of your child's blood samples left over. These samples may be useful for other research studies that have not yet been developed.

If you agree to have your child's samples stored after this study, they will be stored anonymously without your child's name on the samples. The samples will only be used to investigate ways that children and young people respond to HIV and its treatment, for example how the body fights HIV and conditions like tuberculosis which HIV makes people more vulnerable to, and how the HIV virus responds to the body fighting it. The tests may look at cells, proteins, and other chemicals in the body. Tests may also examine your child's genes (DNA), since they might affect how your child's body responds to anti-HIV medicines in important ways. No other kinds of genetic test on your child's stored specimens will be done by anyone, without first explaining the test to you and obtaining your permission.

The study researchers do not plan to contact you, your child or your regular doctor with any results from tests done on your child's stored samples. This is because research tests are often done using experimental tests, so the results may not help to make decisions on managing your child's health. In the very rare case that a specific test result gives important information about your child's health, the researchers will tell the study staff and the study staff will try to contact you. If you wish to be contacted with this type of test result, you must let the study staff know of any change to your contact information.

If your child has a regular doctor and you want the study staff to tell this doctor your child's test results, you must give the study staff your child's doctor's contact information.

Allowing your child's samples to be stored after this study has ended and used in the future is completely voluntary. You may decide not to have any samples stored other than what is needed to complete this study and your child can still be in this research study or any future study.

Even if you decide now that your child's samples can be stored for future research, you may change your mind at any time. If this happens, you must tell the study staff that you have changed your mind. If you decide not to have your child's samples stored and used for future research after this study has ended, any samples remaining in the study laboratory will be destroyed at the end of the study.

The total amount of blood taken will never exceed a safe limit and will be between one and a half and five teaspoons depending on the weight of your child.

We would like your agreement that we can keep the stored samples anonymously (without your child's name or full date of birth) for up to five years after the end of the trial.

Genetic testing

A genetic test is one carried out on DNA from a blood sample. Genes are made up of DNA which are the "messages" making up the instructions which the body uses to do things. The DNA in your genes determines your physical characteristics, everything from your height, to your chance of getting different diseases, to how your body responds to different medicines. We inherit genes from our parents and the unique combination of the genes we have controls the way our body works.

Some genes are known to affect how anti-HIV medicines work in the body and how long they fight the virus for. Others affect how the immune system responds to HIV and to anti-HIV medicines. Other genetic factors may also play an important role, but these are not yet known. Being able to compare the results of genetic tests with the levels of DTG/3TC in the blood and how the immune system copes with the HIV infection and DTG/3TC over time in D3 is very valuable to find out more about these genes.

The tests to identify any such genes are done on samples of blood and involve extracting the DNA, or unique set of genes. If your child is allocated to the DTG/3TC treatment group, the DNA will be extracted at a future date from a special blood sample that is collected from your child at the start of the D3 study.

Because little is known about the importance of any findings from your child's stored DNA, and because these tests will be performed at some date in the future, which is not yet determined, you will not be given results from findings related to this genetic testing. However, you can be assured that any findings from D3 relating to the presence of specific genetic markers that may affect disease or treatment outcome in HIV-infected children and young people will be made available to scientists and doctors to help improve care for children and young people with HIV in the future.

5 What are the possible side-effects?

What are the most common side-effects?

All treatments can have unwanted side effects. The most common side effects are non-severe, and for DTG these are headaches, feeling and being sick, diarrhoea and abdominal pain. 3TC has been used in

children for a long time and it is one of the most well-tolerated anti-HIV medicines. Most children and young people have this or a very similar medicine already included in their treatment regimen. The most commonly reported side effects of 3TC are feeling sick, vomiting, abdominal pain, diarrhoea, rash, joint pains, feeling weak and having a headache.

Are there other side-effects?

Other reported side effects of DTG are rash, itching, feeling light-headed or weak, worrying often, experiencing low mood, sleep problems and abnormal liver function tests (tests which check for liver damage). Some adults and adolescents taking DTG have reported having suicidal thoughts and behaviour; though these may not have been related to this medicine in all cases. If you believe your child is having suicidal thoughts, talk to their study doctor or nurse as soon as possible. There is a small chance that DTG may cause an increase in weight or may cause serious liver problems, which is why the doctor needs to follow your child closely.

It is possible to have an allergic reaction to any medicine. Allergic reactions are uncommon and can range from mild to life-threatening. Symptoms may include skin rash, facial and throat swelling, difficulty breathing, vomiting, light-headedness and racing heartbeat. In severe cases, which are rare, the allergic reaction may involve other organs including the liver, kidneys, lungs, and heart.

If you become concerned about any side-effects, please tell the study staff as soon as possible.

6 What are the possible benefits of taking part in this study?

We hope that your child will be helped by taking part in this study, but we cannot guarantee this. The knowledge learned will definitely help other children and young people with HIV in the future.

If your child is randomised to the DTG/3TC group and they tolerate the medicines well in the first few months, they might get fewer side effects long term, as they are only taking two types of medicine and not three or four.

If your child is randomised to the DTG/3TC group and they weigh 25kg or more they will only have to take one small adult tablet once a day. If they weigh less than 20kg they will take three to six dispersible tablets that can be mixed in a small amount of water. If they weigh 20 to 25kg, they will either take one adult DTG/3TC tablet or six dispersible tablets, depending on which clinic they are treated at (their doctor or nurse will let you know what the option for their clinic is).

If your child is in the control group they will take DTG plus two anti-HIV medicines that are already being given to children and young people. Whichever group your child is in, they will be watched very carefully for any side effects and responses to the treatment.

For most children and young people who are routinely seen every three months, there is only one extra clinic visit (at four weeks after starting in the study).

The information we get from this study will help us to improve treatment for children and young people across the world who are living with HIV and, in future, it may mean that your child has the chance to change to medicines that are easier to take.

7 What are the possible disadvantages and risks of taking part?

Risks in pregnancy and anti-HIV medicines

There is very little information about the effect of DTG in unborn babies. In a recent study from Botswana, approximately two in 1000 participants who were taking DTG when they became pregnant had babies with neural tube defect (a serious brain and/or spine problem). In the same study, approximately one in 1000 participants who were taking other anti-HIV medicines when they became pregnant had babies with neural tube defects, which is similar to what is seen in people without HIV. These problems happen very early in pregnancy, before many people even know that they are pregnant. We do not know for sure if this occurs because of DTG. The study in Botswana and other similar studies are ongoing. We expect more information about this in the near future. In the meantime, we would like to tell you about this possible risk.

If your child has started having their periods, they will need to do a pregnancy test before entering the study. Your child will not be able to join the study if the test shows that they are pregnant. Your child should avoid getting pregnant while in the study and use effective contraceptives if they are having sex. Pregnancy tests will be done at every visit during the study to find out as early as possible if your child is pregnant or not, and to give them the appropriate medical care. If they do become pregnant, they will be able to stay in the study and their doctor will talk to you and your child about whether they need to change their medicine. If your child has a baby whilst participating in the study, the baby will be assessed by a doctor for any problems and will be followed for approximately four weeks to check their health, whereby information such as their HIV status, will be collected.

8 More information about taking part

Does my child have to take part in the D3 study?

No, it is up to you to decide whether or not they should take part. You should talk to them about it if you think they are capable of helping to make the decision – most older children will be able to participate in this discussion even if they cannot legally consent for themselves. If you decide they should take part you will be given this information sheet to keep and you will be asked to sign a consent form.

If you decide for your child to not take part in this study, your child will continue to take the anti-HIV medicines that they are currently on. A decision to not take part at any time will not affect the standard of care that your child receives.

Will I get back any travel costs?

Travel costs for you and your child will be reimbursed.

Can my child stop taking part after we've joined the study?

Your child can stop taking part in all of this study, or in any part of it, at any time and without giving a reason, but you and your child must talk to their study doctor or nurse first. They can advise you about any concerns that either of you may have.

If you or your child decides that they should stop taking the study treatment, we would like to keep your child in the study and continue collecting information about them. This is important because it helps us to ensure that the results of the study are reliable.

If you decide for your child to stop taking part in this study, any data already collected and any stored samples taken while they were in the study will be retained and included in the research analysis. This is to ensure that the results of the study are valid and reliable.

If your child stops taking part in this study, they are likely to receive the standard treatment. A decision to stop taking part at any time will not affect the standard of care your child receives.

How important is it to take the treatments regularly?

It is very important that your child does not miss any doses of anti-HIV medicines and that they do not share their medicines with anyone else.

If your child does not take their anti-HIV medicines as prescribed they may lose their effect, as the HIV virus can become resistant to them. You should ask your child's study doctor or nurse for advice before your child takes any other medicines. If your child does not attend a clinic visit, we may send a home visitor to your home to find out how they are. Please bring your child to the clinic immediately if they take too many pills.

Will my family doctor be informed about my child taking part in this study?

If a doctor in this clinic usually writes a clinic letter to your family doctor or your local doctor after they see your child in the clinic, they will likely include the information in the clinic letter that your child is taking part in the D3 study.

How will my child's personal information be used?

Fondazione Penta Onlus is the sponsor for this study, based in Italy. MRC Clinical Trials Unit at UCL, based in the United Kingdom, will be using information from your child and their medical records in order to undertake this study and will act as a joint data controller for this study. Fondazione Penta Onlus and MRC Clinical Trials Unit at UCL will both be responsible for looking after your child's information and using it properly and will keep identifiable information about your child for 25 years after the study has finished.

Your rights and your child's rights to access, change or move your child's information are limited, as we need to manage your child's information in specific ways in order for the research to be reliable and accurate. If your child withdraws from the study, we will keep the information about your child that we have already obtained. To safeguard your child's rights, we will use the minimum data that can be traced back to your child as possible.

Your child's information (see below) may be sent to ViiV Healthcare Limited, the company providing DTG/3TC and other medicines for the trial, and to regulatory organisations in your country and other

countries, so that DTG/3TC may be licensed for use in other children and young people, if it is shown to work well.

You can find out more about how we use your child's information at **www.ctu.mrc.ac.uk/general/privacy-policy** and **www.penta-id.org/study-participants-privacy**.

How will my child's data be stored and collected?

Your child's clinic will collect information from your child and their medical records for this research study in accordance with our instructions.

Your child's clinic will use your child's name, a health service identification number specific to your country/hospital (for example, NHS number if you live in the UK) and contact details to contact you about the research study, and make sure that relevant information about the study is recorded for your child's care, and to oversee the quality of the study. Your child's clinic will keep your child's name, a health service identification number specific to your country/hospital and contact details confidential and will not pass this information to MRC Clinical Trials Unit at UCL (or the PHPT Clinical Trial Unit for the study sites in Thailand).

MRC Clinical Trials Unit at UCL (and PHPT for the study sites in Thailand) will collect health information about your child for this research study from your child's hospital, which is regarded as a special category of information. We will also collect date of birth, but no other personal identifying information. We will use this information to conduct our research. Authorised individuals from Fondazione Penta Onlus, MRC CTU at UCL, ViiV Healthcare, regulatory organisations and the authorised monitors may look at your medical and research records to check the accuracy of the research study.

Where information could identify your child, the information will be held securely with strict arrangements about who can access the information. The people who analyse the information will not identify your child.

Your child's clinic will keep identifiable information about your child from this study for at least 25 years after the study has finished.

What happens at the end of the study?

You and your child and their doctor will continue to make decisions about your child's medicines once the study has finished. If your child is in the DTG/3TC group and treatment with DTG/3TC appears to be an effective way of treating children and young people with HIV, with few side effects, then they may be able to stay on these medicines. We would like your permission to collect information for 30 days after your child finishes the study.

Future Research

When you agree for your child to take part in a research study, the information about their health and care may be provided to researchers running other research studies. This is to maximise the amount of good science that can be done through people participating in the study. These other studies may be run by universities, National Health Service organisations or companies involved in health and care research in this country or abroad. Your child's information will only be used by organisations and researchers to conduct research in accordance with relevant legislation, ethics and National Health Service research policy requirements.

We won't share information that can identify your child to others. The information will only be used for the purpose of health and care research, and cannot be used to contact your child or affect their care. It will not be used to make decisions about future services available to you, such as insurance. If there is a risk that your child can be identified, their data will only be used in research that has been independently reviewed by an ethics committee.

What will happen to the results of the D3 study?

When the study is completed, we will publish a summary of the results on the website of the MRC CTU at UCL (http://www.ctu.mrc.ac.uk/) and Fondazione Penta Onlus website (https://penta-id.org/).

We will also tell all the participants in the study, and their carers, the results of the study. We may do this in a short information sheet, or through community meetings held at your child's clinic.

We will also publish the results in a medical journal, so that other doctors can see them. You can ask your child's doctor for a copy of any publication. Your child's identity and any personal details will be kept confidential. No named information about your child will be published in any report of this study.

Who is organising and funding the study?

This study is organised by Fondazione Penta Onlus and the MRC CTU at UCL, which have run trials for many years. The study coordination, data collection and analysis and administration will be provided by the MRC CTU at UCL, and at the study sites in Thailand, by PHPT. You can find out more about us at www.ctu.mrc.ac.uk/. The study is funded by ViiV Healthcare.

Your child's doctor is not receiving any money or other payment for asking your child to be part of the study.

Fondazione Penta Onlus has overall responsibility for the conduct of the study. They are responsible for ensuring the study is carried out ethically and in the best interests of the study participants.

Who has reviewed the D3 study?

The study has been reviewed by international scientists and clinicians. It has been approved by MRC CTU at UCL, Fondazione Penta Onlus, and ViiV Healthcare Ltd.

It has been authorised by the [insert Regulatory Agency specific to the country, e.g. in the UK: Medicines and Healthcare products Regulatory Agency (MHRA)], as well as by [insert name of research ethics committee] and the hospital's Research and Development Office.

What if new information becomes available during the study?

Sometimes during a study, new information becomes available about the medicines that are being studied. If this happens, your child's doctor will tell you about it and discuss with you whether you want your child to continue in the study. If you decide for your child to stop taking part in the study, your child's doctor will arrange for their care to continue outside of the study.

Your child's doctor might also suggest that it is in your child's best interests to stop taking part in the study. Your child's doctor will explain the reasons and arrange for your child's care to continue outside the study.

What happens if the D3 study stops early?

Very occasionally a study needs to be stopped early. If this happens, the reasons will be explained to you and your child, and your child's doctor will arrange for your child's care to continue outside of the study.

What if something goes wrong for my child?

If you have any concerns about the way you or your child have been approached or treated during the study, please talk to your child's study doctor or nurse. If you are still unhappy, or if you wish to complain, please use the normal clinic or hospital complaints process.

If your child is harmed by taking part in the study, or if your child is harmed because of someone's negligence, then you may be able to take legal action.

9 Contacts for further information

If you want further information about the D3 study, contact your study doctor or nurse (see below).

[Insert address and telephone number of study doctor and/or nurse]

More information is also available on our website https://penta-id.org/hiv/d3-penta-21/.

Thank you for taking the time to consider taking part in this study.

To be presented on local headed paper To be presented on local headed paper

[Remove text box and insert relevant logos here]

Version 3.0 Date 04-October-2021

D3 (Penta 21): A randomised non-inferiority trial with nested PK to assess DTG/3TC fixed dose formulations for the maintenance of virological suppression in children with HIV infection aged 2-<15 years old

Centre Name: …………………………………………………..……………..

Study Number: ………………………………………..……………………..

Study 3-Letter Code: …………………………………….……………………

Name of Researcher: ……………………………………………..……………

Please initial each box (or mark with your thumb) if you agree:

1. I confirm that I have read/ been read the patient information sheet (Version 3.0 Date 04-October-2021) for the D3 study and agree to my child being assessed to see whether they are eligible to take part in this study. I understand what will be required if my child participates in the study.	
2. The D3 study has been explained to me and my questions have been answered.	
3. I understand that the results of the screening assessments might show that my child is not eligible to take part in the D3 study.	
4. I understand that my child's participation is voluntary and that I am free to withdraw them at any time, without giving any reason, without my medical care or legal rights or my child's medical care or legal rights being affected.	
5. I understand that sections of any of my child's medical notes may be looked at by responsible individuals involved in the running of the study, or by regulatory authorities or ViiV Healthcare, the company providing the new medicine for this study, where it is relevant to my child's participation in this research. I give permission for these individuals to have access to my child's records, but understand that strict confidentiality will be maintained.	
6. I agree to allow blood samples to be taken from my child and analysed for the purpose of the D3 study. I give permission for these samples to be tested in a laboratory in a different country, if required.	
7. I agree to routine blood results and clinical information being included anonymously in continued follow-up after my child's last study visit (identified only by study number, date of birth and random letter code).	

Please turn over the page

8. I agree to UCL collecting information about my child from my child's clinic for this research study, including date of birth. This data may be used by UCL, Penta and other authorised research collaborators to conduct the trial and associated sub-studies. I understand that this will involve my child's anonymised information being analysed in another country.	
9. I agree to anonymised study data being transferred to ViiV Healthcare, and to regulatory agencies in this country and other countries.	
10. I agree for my child to participate in the D3 study if the screening tests show that they are eligible.	
11. I understand that if I am unable to continue to be the main carer for the child that I need to provide the clinic with the name of the person who will become the main carer so their consent can be requested.	
12. I agree to allow a blood sample to be taken from my child to test for genetic markers to help to understand how genes affect response to treatment. I understand that my child and I may not be given the results of these tests. *(If you do not wish to give this permission, do not put your initials or thumb mark in the box – your child can still take part in the study).*	
13. I agree to allow my child's data to be made available for research outside of the D3 study, where the data would be stored appropriately and the research approved separately. I understand that I may not be given the results of these research studies. *(If you do not wish to give this permission, do not put your initials or thumb mark in the box – your child can still take part in the study).*	
14. I agree to allow my child's blood samples to be stored for future research outside of the D3 study, where the samples would be stored appropriately and the research approved separately. I understand that these samples will not be identified by either my or my child's name and I may not be given the results of tests performed on stored samples. *(If you do not wish to give this permission, do not put your initials or thumb mark in the box – your child can still take part in the study. Note, future research on samples would require use of your child's data, as detailed in point 13 above).*	

If you agree that your child may take part, please complete the section below:

Parent/carer's signature (or thumbprint)	Print name	Date
Witness's signature (if thumbprint used above)	Print name	Date

Please turn over the page

The person who explained the study to you needs to sign too:

I have provided the D3 study information to the participant's parent/legal guardian in full and answered all of their questions. To the best of my knowledge, they understand the purpose, interventions, risks and benefits of this study and willingly agree for their child to be enrolled into the D3 study.		
Signature of person conducting the informed consent process	Print name	Date

I would/would not *(please circle)* like my child's family doctor to be notified about my child's participation in this study.
[Country specific, delete if not applicable]

Signature of parent/guardian: ________________________________ Date: ____________

Name of family doctor: ___________________________________

Contact address of family doctor: ____

IMPORTANT: **one signed original to be kept in D3 trial file by the researcher**
one signed copy to be given to the Parent/Guardian
one signed copy to be kept in the clinic file
